# Supplementary material for: Antibody-Directed Glucocorticoid Targeting to CD163 in M2-type Macrophages Attenuates Fructose-Induced Liver Inflammatory Changes
Source: Mol Ther Methods Clin Dev. 2016 Dec 24;4:50–61. doi: 10.1016/j.omtm.2016.11.004 (PMC5363319; doi:10.1016/j.omtm.2016.11.004)
Supplement: Document S1. Supplemental Materials and Methods, Figures S1–S2, and Tables S1–S4 [file mmc1.pdf]

**OMTM, Volume *4***

## Supplemental Information

# Antibody-Directed Glucocorticoid Targeting to CD163 in M2-type Macrophages Attenuates Fructose-Induced Liver Inflammatory Changes

**Pia Svendsen, Jonas H. Graversen, Anders Etzerodt, Henrik Hager, Rasmus Røge, Henning Grønbæk, Erik I. Christensen, Holger J. Møller, Hendrik Vilstrup, and Søren K. Moestrup**

# Supplementary Material

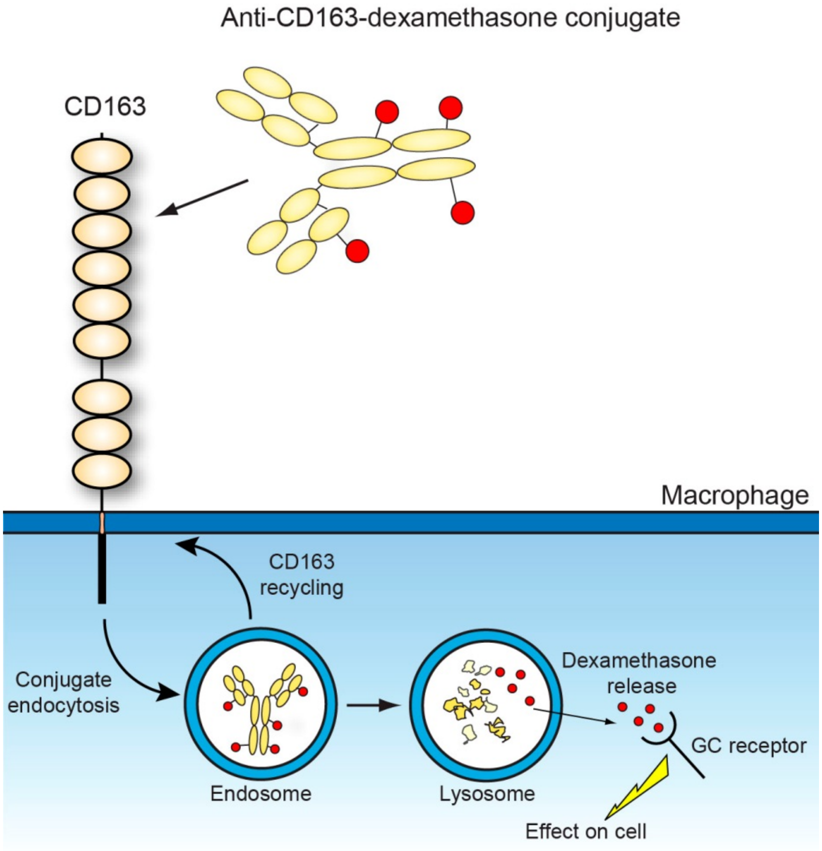


**Figure S1.** Mechanism of action of anti-CD163 IgG-dexamethasone conjugates. The conjugate that in average contains four dexamethasone drug molecules per IgG protein linked with a breakable ester bonds binds to the haptoglobin-hemoglobin receptor, CD163. This macrophage-specific receptor mediates a fast endocytosis leading to lysosomal degradation of the anti-CD163-dexamethasone conjugate. Dexamethasone now released can diffuse into the cytosol where the drug effect is achieved by binding to the cytosolic GC receptor.

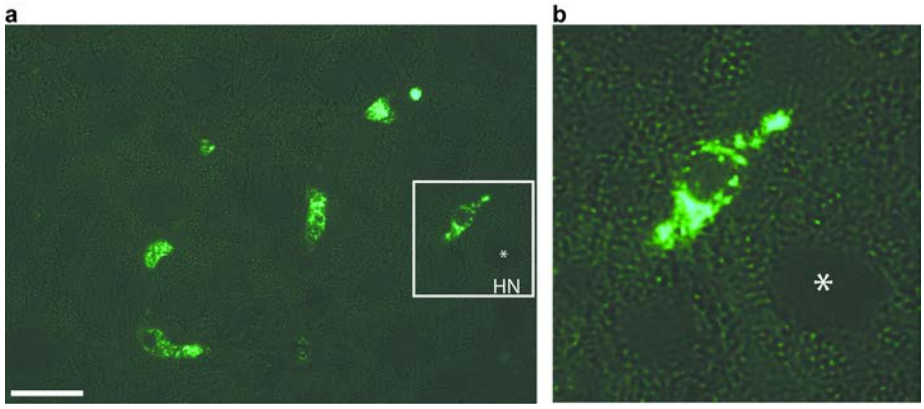


**Figure S2.** Fluorescence microscopic bright-field analysis of formalin-fixed cryosection of the rat liver one-hour post i.v. injection of the anti-CD163-dexamethasone conjugate. Anti-mouse IgG-Alexa Fluor 488 (green) was used for detection of the conjugate. (b) Enlargement of area indicated in (a) showing vesicular staining in accordance with uptake in Kupffer cell. White scale bar equals 20. *indicates hepatotocyte cell nucleus(HN).

**Table S1.** Liver mRNA profiling of NASH associated genes significantly up- or down-regulated above a 1.4 fold level in rats feed on HFr diet compared to STD.

| Pathway/response | Gene symbol | Encoded protein | Fold Regulation | P-value |
|---|---|---|---|---|
| **Carbohydrate metabolism** | | | | |
| | *Gk* | Glycerol kinase | -2.1 | 0.005 |
| | *G6pd* | Glucose-6-phosphatase, catalytic subunit | 5.2 | 0.029 |
| | *Pklr* | Pyruvate kinase, liver and red blood cell | 2.1 | 0.003 |
| | *Rbp4* | Retinol binding protein 4, plasma | -1.5 | 0.005 |
| **Lipid and fatty acid metabolism** | | | | |
| | *Acaca* | Acetyl-coenzyme A carboxylase | 2.9 | 0.002 |
| | *Acsl5* | Acyl-CoA synthetase long-chain family member 5 | 3.1 | 0.001 |
| | *Acly* | ATP citrate lyase | 4.9 | 0.031 |
| | *Acsm3* | Acyl-CoA synthetase medium-chain family member 3 | -4.5 | 0.006 |
| | *Fabp5* | Fatty acid binding protein 5, epidermal | 3.0 | 0.031 |
| | *Fasn* | Fatty acid synthase | 6.4 | 0.006 |
| | *Lpl* | Lipoprotein lipase | -2.7 | 0.002 |
| | *Scd1* | Stearoyl-Coenzyme A desaturase 1 | 5.8 | 0.003 |
| **Cholesterol metabolism/pathway** | | | | |
| | *Abca1* | ATP-binding cassette, sub-family A (ABC1), member 1 | -1.5 | 0.010 |
| | *Cyp2e1* | Cytochrome P450, family 2, subfamily e, polypeptide 1 | -2.3 | 0.001 |
| | *Nr1h2* | Nuclear receptor subfamily 1, group H, member 2 | -1.6 | 0.024 |
| | *Nr1h4* | Nuclear receptor subfamily 1, group H, member 4 | -1.4 | 0.045 |
| | *Srebf2* | Sterol regulatory element binding factor 2 | -1.4 | 0.021 |
| **Oxidative phosphorylation** | | | | |
| | *Ppa1* | Pyrophosphatase (inorganic) | -1.6 | 0.008 |
| **Adipokine Signaling** | | | | |
| | *Adipor1* | Adiponectin receptor 1 | -1.6 | 0.010 |
| | *Rbp4* | Retinol binding protein 4 | -1.5 | 0.005 |
| **Insulin Signaling** | | | | |
| | *Pklr* | Pyruvate kinase (liver ) | 2.1 | 0.003 |
| | *Igf1* | Insulin-like growth factor 1 | -1.7 | 0.011 |

**Table S2.** Cytokine levels in LPS challenged rats (n=4 per group).

| Treatment group | TNF (ng/ml) 1 hour post LPS challenge | IL-6 (ng/ml) 1 hour post LPS challenge |
|---|---|---|
| **Vehicle** | 7.43 (0.65) | 23.9 (2.1) |
| **Dexamethasone (1 mg/kg)** | 3.27 (1.50)* | 13.7 (6.4) |
| **Dexamethasone (0.02 mg/kg)** | 4.18 (1.61) | 17.5 (5.7) |
| **Anti-CD163-dexamethasone (0.02 mg/kg)** | 0.64 (0.33)** | 7.0 (4.8)** |
| **Control IgG-dexamethasone (0.02 mg/kg)** | 5.18 (1.18) | 17.4 (3.9) |
| **Anti-CD163** | 7.45 (0.96) | 30.0 (0.6) |

Values are means ±SEM. Significant differences between the vehicle group and the treatment groups are indicated by *$p<0.05$, **$p<0.01$.

**Table S3.** Food intake in treatment groups during the NASH treatment study.

| Treatment group | HrF intake (g/day/cage*) |
|---|---|
| **Vehicle** | 35.76 (1.03) |
| **Dexamethasone (0.02 mg/kg)** | 35.40 (0.94) |
| **Anti-CD163-dexamethasone (0.02 mg/kg)** | 35.40 (0.79) |
| **Control IgG-dexamethasone (0.02 mg/kg)** | 36.00 (0.77) |

Values are means ±SEM. The food intake was monitored during the entire experiment, but no significant differences were observed between the treatment groups. * Two rats per cage.

**Table S4.** Liver RNA profiling of genes significantly up- or down-regulated above a 1.4 fold threshold level in a treatment group(s) compared to the vehicle group. Fructose-induced up ( ↑ ) or down ( ↓ )-regulation of those genes are indicated in the right side column.

| Treatment group | | | Dexa | Anti-CD163-dexa | IgG-dexa | HFr-induced change |
|---|---|---|---|---|---|---|
| **Pathway/ response** | Gene symbol | Encoded protein | Fold Regulation | Fold Regulation | Fold Regulation | Up-or down Regulation |
| **Carbohydrate metabolism** | | | | | | |
| | *Gk* | Glycerol kinase | 1.5* | 1.7* | 1.9** | ↓** |
| **Lipid and fatty acid metabolism** | | | | | | |
| | *Acly*[A] | ATP citrate lyase | 1.2 | -1.4* | -1.2 | ↑** |
| | *Acsm3* | Acyl-CoA synthetase medium-chain family member 3 | -1.8* | -1.3 | -1.0 | ↓** |
| | *Slc27a5* | Solute carrier family 27, member 5 | -1.1 | 1.3* | 1.5** | ↓* |
| **Cholesterol metabolism/transport** | | | | | | |
| | *Apoa1* | Apolipoprotein A-I | 1.4 | 1.3 | 1.5* | ↓* |
| | *Ldlr* | Low density lipoprotein receptor | 1.2 | 1.2 | 1.4* | NS |
| | *Nr1h3* | Nuclear receptor subfamily 1, group H, member 3 | -1.2 | -1.4** | -1.3 | NS |
| | *Ppara*[A] | Peroxisome proliferator activated receptor-α | 1.0 | 1.3 | 1.6** | ↓** |
| | *Ppard*[A] | Peroxisome proliferator activated receptor-δ | 1.2 | -1.3 | -1.9** | NS |
| | *Pparg*[A] | Peroxisome proliferator activated receptor-γ | -1.6* | -1.4 | -13.5** | ↓** |
| **Mitochrondial β-oxidation** | | | | | | |
| | *Acadl* | Acetyl-Coenzyme A dehydrogenase, long-chain | 1.3 | 1.6*** | 1.3 | ↓* |
| | *Cpt1a* | Carnitine palmitoyltransferase 1a, liver | 1.5 | 2.4*** | 2.8** | ↓* |
| **Adipokine signaling** | | | | | | |
| | *Lepr*[A] | Leptin receptor | 1.3 | 1.5** | 1.6** | NS |
| **Insulin Signaling** | | | | | | |
| | *Insr* | Insulin receptor | 1.2 | 1.3 | 1.4* | ↓** |
| | *Ptpn1* | Protein tyrosine phosphatase, non-receptor type 1 | -1.1 | -1.3 | -1.7* | NS |
| **Inflammation** | | | | | | |
| | *Cebpb*[A] | CCAAT/enhancer binding protein (C/EBP), β | -1.1 | -2.1** | -2.1** | NS |
| | *CD163* | Hemoglobin scavenger receptor, CD163 | -1.026 | -1.236 | -1.6* | ↓* |
| **Apoptosis/Fibrogenesis** | | | | | | |
| | *Casp3* | *Caspase 3, apoptosis-related cysteine protease* | 1.4 | 1.5*** | 1.5** | NS |
| | *Mapk8*[A] | *Mitogen-activated protein kinase 8* | 1.8 | 1.8* | 1.8 | NS |
| | *Ifng*[A] | *Interferon-γ* | -38.1* | 21.6 | -14.3 | NS |
| | *Serpine*[A] | *Serine peptidase inhibitor, clade E, member 1* | -4.8 | -5.9** | -3.3* | NS |

Values are means ±SEM (n=8 per group). Significant differences are indicated by *p<0.05, **p<0.01-0.001. [A] Genes involved in several pathways/responses. NS indicates non-significant regulation.

**Supplementary Material and Methods**

*The rat LPS model.*
The experimental procedures were performed as described (27).

*Fluorescence microscopy.*
The experiment was performed as described in MATERIAL and METHODS (fluorescence microscopy).

*RNA Profiling.*

Total RNA was purified from liver using the RNeasy Mini Kit (Qiagen, København Ø, Denmark). A RT² Profiler™ PCR Array with 92 NASH biomarkers was designed for Fluidigm® BioMark™ analysis (see Supplementary Excel file for target and primer details). Reverse-transcription of RNA and preamplification of genes of interest was done using the Fluidigm Reverse Transcription Master Mix and the Fluidigm PreAmp Master Mix (Fluidigm Corporation) in accordance with the manufactures instructions. In short, for initial cDNA synthesis 25 ng of RNA was mixed with the RT mastermix (5x) in a 1:1 ratio and diluted 5 times in RNAse-free $H_2O$ followed by incubation at the following conditions: 25°C for 5 min, 42°C for 30 min and finally 85°C for 5 min. To increase sensitivity, genes were pre-amplified by pooling 96 deltagene assays (Fluidigm, see Supplementary Excel for full list of target and primer details) allowing a final concentration of 500 nM per assay. In the final pre-amplification reaction mixture cDNA was diluted 1:4 with Fluidigm PreAmp Master Mix (5X), pooled deltagene assays (10X) and $H_2O$. Reaction mixture was incubated at the following PCR conditions: 95°C for 2 min followed by 14 cycles of 95°C for 15 sec and 60°C for 4 min. To remove unincorporated primers pre-amplified cDNA was incubated with 1,25U/µl Exonuclease I at 37°C for 30 min followed by 80°C for 15 min. After Exonuclease I treatment pre-amplified cDNA was diluted 1:5 with TE buffer (10 mM Tris-HCl, 1 mM disodium EDTA, pH 8.0) and stored at -20°C. Gene expression analysis was carried out using the 96.96 dynamic arrays and Biomark HD system from Fluidigm (Fluidigm Europe B.V.). Specifically, a 6 µl sample mixture was prepared for each sample containing 1x SsoFast EvaGreen Supermix with low ROX (BioRad), 1x DNA Binding Dye Sample Loading Reagent and each of diluted pre-amplified samples. Six µl of Assay mix was prepared with 1x Assay Loading Reagent, 100 µM of each of the different Deltagene Assays and TE buffer. The 96.96 dynamic arrays were primed and loaded in an IFC controller with in between additions of samples and assay mixes in the appropriate inlets. After loading, the chip was placed in the BioMark HD Instrument for an initial thermal mix that included incubation at 70°C for 40 min followed by 60°C for 0.5 min. Subsequent qPCR was performed by 1 cycle of hot start at 95°C for 1 min, followed by 30 cycles at 96° C for 5 sec and 60°C for 20 sec with fluorescent recording after each cycle. For analysis of PCR products the qPCR run was followed by a melting curve analysis where the fluorescent signal from dsDNA was measured at 60°C-95°C with 1°C increment. The data was analyzed with Real-Time PCR Analysis Software (Fluidigm Europe B.V.). The RT2 Profiler PCR Array Data Analysis Webportal (www.sabioscience.com) was used to analyze CT values and to calculate changes in gene expression. The relative expression levels of each target gene was normalized to ribosomal protein L13A and beta-actin and calculated by the 2-delta delta Ct method.
