# Supplementary material for: Antibody-Directed Glucocorticoid Targeting to CD163 in M2-type Macrophages Attenuates Fructose-Induced Liver Inflammatory Changes
Source: Mol Ther Methods Clin Dev. 2016 Dec 24;4:50–61. doi: 10.1016/j.omtm.2016.11.004 (PMC5363319; doi:10.1016/j.omtm.2016.11.004)
Supplement: Document S2. Article plus Supplemental Information [file mmc3.pdf]

Molecular Therapy
Methods & Clinical Development
Original Article

Antibody-Directed Glucocorticoid Targeting to CD163 in M2-type Macrophages Attenuates Fructose-Induced Liver Inflammatory Changes

Pia Svendsen,[1] Jonas H. Graversen,[1] Anders Etzerodt,[2,3] Henrik Hager,[4] Rasmus Røge,[5] Henning Grønbæk,[6] Erik I. Christensen,[2] Holger J. Møller,[7] Hendrik Vilstrup,[6] and Søren K. Moestrup[1,2,7,8]

[1]Department of Molecular Medicine, University of Southern Denmark, 5000 Odense, Denmark; [2]Department of Biomedicine, Aarhus University, 8000 Aarhus, Denmark; [3]Centre d'Immunologie de Marseille-Luminy (CIML), 13009 Marseille, France; [4]Department of Pathology, Vejle Hospital, University of Southern Denmark, 5000 Odense, Denmark; [5]Department of Pathology, Aalborg University Hospital, 9100 Aalborg, Denmark; [6]Department of Hepatology, Aarhus University Hospital, 8000 Aarhus, Denmark; [7]Department of Clinical Biochemistry, Aarhus University Hospital, 8000 Aarhus, Denmark; [8]Department of Clinical Biochemistry and Pharmacology, Odense University Hospital, 5000 Odense, Denmark

Increased consumption of high-caloric carbohydrates contributes substantially to endemic non-alcoholic fatty liver disease in humans, covering a histological spectrum from fatty liver to steatohepatitis. Hypercaloric intake and lipogenetic effects of fructose and endotoxin-driven activation of liver macrophages are suggested to be essential to disease progression. In the present study, we show that a low dose of an anti-CD163-IgG-dexamethasone conjugate targeting the hemoglobin scavenger receptor CD163 in Kupffer cells and other M2-type macrophages has a profound effect on liver inflammatory changes in rats on a high-fructose diet. The diet induced severe non-alcoholic steatohepatitis (NASH)-like changes within a few weeks but the antibody-drug conjugate strongly reduced inflammation, hepatocyte ballooning, fibrosis, and glycogen deposition. Non-conjugated dexamethasone or dexamethasone conjugated to a control IgG did not have this effect but instead exacerbated liver lipid accumulation. The low-dose anti-CD163-IgG-dexamethasone conjugate displayed no apparent systemic side effects. In conclusion, macrophage targeting by antibody-directed anti-inflammatory low-dose glucocorticoid therapy seems to be a promising approach for safe treatment of fructose-induced liver inflammation.

INTRODUCTION

Non-alcoholic fatty liver disease (NAFLD) is the most common chronic liver disease[1] and its incidence has increased globally.[2–4] NAFLD and its more progressed form, non-alcoholic steatohepatitis (NASH) with inflammation and fibrosis of the liver,[5–7] can be induced experimentally in rodents by high-fat and/or high-carbohydrate feeding.[8–10] A multi-hit hypothesis is widely accepted as the pathogenic model of disease progression, in which primary hits (i.e., insulin resistance and intrahepatic fat accumulation) promote development of steatosis, sensitizing the liver to secondary hits (e.g., endotoxin exposure, pro-inflammatory cytokine imbalance, and oxidative stress).[11,12] Studies of animal models of NAFLD/NASH have shown that liver macrophage (Kupffer cell) activity has an important triggering role in development of the disease.[13,14]

Special attention is given to dietary fructose, partly because the prevalence of NAFLD in humans has increased in parallel with the consumption of fructose. Furthermore, fructose is far more effective in inducing experimental NAFLD than glucose.[8] The pathogenic mechanisms leading to fructose-induced NAFLD are not fully understood, but it seems evident that the different metabolism and metabolic effects of fructose compared to glucose add to the general effects of hypercaloric intake.[15,16] In brief, fructose in large quantities is almost entirely metabolized in the liver, where it in an insulin-independent way is converted into fructose-1-phosphate and triose-phosphates. The essential fructokinase and aldolase B enzymes involved are not regulated by hepatocyte energy status. Consequently, virtually all ingested fructose is metabolized to triose-phosphates in the liver, where these intermediates are oxidized and converted to glucose, lactate, or triglyceride (TG). TG formation by hepatic de novo lipogenesis contributes to increased hepatic lipid accumulation and plasma TG levels.

To date, the gold standard for treatment of NAFLD consists of a lifestyle modification program aimed to achieve gradual weight loss through dietary changes and physical exercise. However, medical intervention to reverse the condition is desirable for individuals with progressed inflammatory disease.[4]

Therapeutic agents with antioxidant effects such as vitamin E and insulin-sensitizing substances such as thioglitazones, metformin, glucagon-like peptide-1 receptor agonists (GLP-1 RAs), and dipeptidyl peptidase-4 inhibitors have been evaluated for treatment of

Received 7 September 2016; accepted 15 November 2016;
http://dx.doi.org/10.1016/j.omtm.2016.11.004.

Correspondence: Søren K. Moestrup, Department of Molecular Medicine, University of Southern Denmark, J.B. Winsløws Vej 25, 5000 Odense C, Denmark.
E-mail: smoestrup@health.sdu.dk

 © 2016 The Author(s).
This is an open access article under the CC BY license (http://creativecommons.org/licenses/by/4.0/).

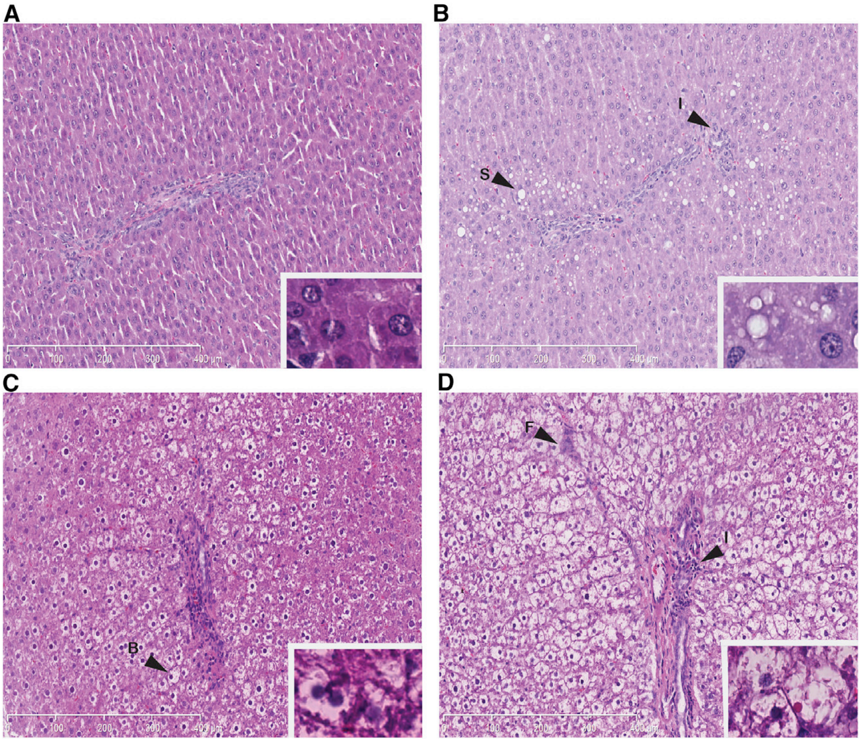

Figure 1. Steatosis, Infiltration of Inflammatory Cells, and Hepatocyte Ballooning in Rats on the HFr Diet

Histological changes examined by H&E staining (×10) of rat liver after (A) 2 weeks on the STD diet, (B) 2 weeks on the HFr diet, (C) 5 weeks on the HFr diet, or (D) 12 weeks on the HFr diet. Scale bars represent 400 μm. Insets of higher magnification ×40 are shown in the lower right corner. Arrowheads indicate areas of steatosis (S), leukocyte infiltration (I), hepatocyte ballooning (B), and fibrosis (F).

NASH.[17–19] The latter two classes of pharmacotherapies,[20,21] collectively designated incretin-based therapies, seem most promising, but to date no efficient drugs have been approved for NASH.[22,23] Glucocorticoids (GCs) may dampen progression of inflammation but they are contraindicated because of their profound and well-known side effects that may even induce NAFLD/NASH in liver hepatocytes.[24] Low-dose GCs are a medical treatment option for acute alcohol-induced steatohepatitis, although their marginal effect is a matter of discussion.[25,26]

We therefore decided to investigate whether molecular targeting of macrophages (and thereby bypassing hepatocytes) with a low-dose GC may deliver GC at therapeutic doses to Kupffer cells and thereby dampen fructose-induced steatohepatitis progression without adverse GC effects. For this study, we used a recently developed anti-inflammatory antibody-drug conjugate (ADC) (Figure S1) composed of the synthetic GC dexamethasone (dexa)[27,28] linked to an antibody against the macrophage receptor CD163. This drug conjugate exhibits a strong dose-dependent reducing effect on endotoxin-induced cytokine release in rats and pigs.[27,29] Compared to free dexamethasone, potency of the CD163-targeted dexamethasone is 50-fold stronger.[27,29] CD163, which functions as the scavenger receptor[30] of haptoglobin-hemoglobin complexes[31,32] and other ligands,[28,33] is highly upregulated in M2-type macrophages, including liver Kupffer cells (main macrophage subset), and in infiltrating macrophages in sites of inflammation.[28,34] CD163 is a constitutive recycling endocytic receptor for transport of cargo to lysosomes rather than a receptor for intracellular signal transmission.[28] This function, combined with its selected and high expression in macrophages, makes it an ideal macrophage target for drug delivery.

RESULTS

Development of NAFLD/NASH in Rats Fed a High-Fructose Diet

Two weeks after initiation of a high-fructose (HFr) diet, the livers of Sprague-Dawley rats exhibited morphological signs of steatosis with fat accumulation in hepatocytes (Figure 1B). The steatosis at this stage was predominantly seen as microvesicular fat infiltration with a component of macrovesicular steatosis displacing the central hepatocyte nucleus. After 5 weeks, hepatocyte ballooning was observed as a morphological feature of early steatohepatitis (Figure 1C). Increased hepatocyte ballooning, along with other histological lesions such as Mallory bodies and glycogenated nuclei, appeared after 12 weeks in rats fed an HFr diet (Figure 1D; Table 1). Inflammatory infiltrates with leukocytes were initially observed after 2 weeks and they markedly increased over time (Figures 1B–1D). The development of liver fibrosis was evident after 5 weeks (Figure 1C). Fibrosis was characterized by both pericellular and perivenular deposition, and advanced fibrosis with a distinct chicken-wire bridging pattern was seen after 12 weeks (Figure 1D). Immunostaining of macrophages revealed that the population of CD163-positive cells (Figures 2A and 2B) was also increased after 2 weeks ($p = 0.0019$), thus showing that the Kupffer cell population is supplemented with infiltrating M2-type macrophages.

Liver/body weight ratios were highly elevated after 12 weeks in rats on the HFr diet compared to a standard (STD) diet (Table 1). Body weight differences between the two groups were not seen until after 6 weeks on the HFr diet; however, from 6 to 12 weeks, the body weight of rats on the HFr diet was significantly higher compared to rats on the STD diet ($p = 0.026$). After 2 weeks, serum levels of glucose as well as aspartate transaminase (AST) activity were higher in rats on the HFr diet compared to control rats on the STD diet. The serum concentration of glucose, cholesterol, and triglyceride increased over time (Table 1). Furthermore, the level of serum tumor necrosis factor alpha (TNF-α) was elevated after 2 and 12 weeks; after

Table 1. Organ Weight, Histological Scoring, and Paraclinical Characteristics of Rats Fed the HFr Diet

Parameter	STD (n = 4) 2 weeks	HFr (n = 4) 2 weeks	STD (n = 8) 12 weeks	HFr (n = 8) 12 weeks
Body weight (g)	260.6 ± 8.2	257.8 ± 3.6	454.8 ± 10.0	483.2 ± 7.2*
Liver/body weight (g/100 g)	3.4 ± 0.06	3.5 ± 0.11	3.6 ± 0.05	4.6 ± 0.11**
Spleen/body weight (g/100 g)			0.22 ± 0.01	0.21 ± 0.01
Steatosis (0–3)	0	1.25 ± 0.25**	0	3.00 ± 0.13**
Inflammation (0–3)	0	0.75 ± 0.25*	0	2.13 ± 0.25**
Hepatocyte ballooning (0–2)	0	0	0	1.75 ± 0.14**
Fibrosis (0–8)	0	0	0	5.25 ± 0.65**
Pericellular (0–4)			0	2.25 ± 0.25**
Perivenular (0–4)			0	3.00 ± 0.40**
Glycogen deposition (0–4)			0.25 ± 0.16	2.75 ± 0.31**
ALT (U/L)	43.4 ± 5.6	43.4 ± 2.7	66.4 ± 10.4	83.9 ± 16.4
AST (U/L)	136.3 ± 3.0	211.6 ± 19.4**	117.3 ± 11.7	134.6 ± 31.9
ALP (mM)	243.5 ± 17.1	151.9 ± 9.2**	188.3 ± 15.3	133.8 ± 3.7*
Glucose (mM)	4.8 ± 0.5	6.3 ± 0.5*	5.0 ± 0.2	8.5 ± 0.8**
Cholesterol (mM)	2.4 ± 0.1	2.4 ± 0.1	2.0 ± 0.2	2.6 ± 0.1*
HDL (mM)			1.4 ± 0.1	1.2 ± 0.1
Triglyceride (mM)	1.3 ± 0.1	1.0 ± 0.1*	1.5 ± 0.6	3.7 ± 0.5**
Creatinine (mM)			23.0 ± 1.5	17.5 ± 0.9*
Phosphate(mM)			1.6 ± 0.1	2.1 ± 0.1*
TNF-α (pg/mL)	<ND	20.6 ± 9.4**	0.2 ± 0.05	11.0 ± 1.2**
IL-1β (pg/mL)	<ND	9.4 ± 4.6	0.2 ± 0.03	11.5 ± 1.9**

Values are means ± SEM. Significant differences between the control groups on the STD diet and the HFr diet after 2 and 12 weeks are indicated by *$p < 0.05$ and **$p < 0.01$, respectively.

12 weeks, both the levels of TNF-α and interleukin (IL)-1β were significantly higher in rats on the HFr diet compared to rats on the STD diet (Table 1). In accordance with these fructose-induced NASH-like effects, hepatic mRNA profiling of rats after 12 weeks on the HFr diet revealed marked changes in the transcription level of multiple genes involved in metabolism of carbohydrates, fatty acids, and cholesterol as well as genes involved in adipokine and insulin signaling (Table S1).

In conclusion, initial stages of steatosis and inflammation were seen after 2 weeks and pronounced disease progression with fibrosis developed during the 12-week study period (Table 1). These histopathological changes were accompanied by fructose-induced production of pro-inflammatory cytokines as well as a changed regulation of several metabolic pathways involved in the development of NASH.[35–38] This included upregulation of genes critical for synthesis, activation, and oxidation of fatty acids and downregulation of genes that contribute to intra- and extracellular cholesterol homeostasis[39–41] as well as insulin resistance and signaling, respectively.[42,43] The treatment effect of targeting Kupffer cells with anti-CD163-dexamethasone was subsequently investigated in rats that developed HFr-diet-induced liver disease.

Targeting of the Anti-CD163-Dexamethasone Conjugate to Liver Macrophages

The anti-rat CD163 monoclonal antibody designated "ED2" used in the following studies of drug targeting to CD163 has been shown to target only macrophages[44] and to have a plasma half-life of a few minutes, owing to fast uptake in macrophage-rich organs (particularly the liver, which accounts for the majority of the uptake).[27,45] Fluorescence microscopy of liver sections from rats that were on a standard diet and were injected intravenously (i.v.) with the ED2-dexamethasone conjugate accordingly demonstrated specific vesicular uptake of this anti-CD163-dexamethasone conjugate in single cells with a Kupffer cell-like shape and distribution (Figures 2C and S2), whereas only weak non-specific staining was observed in rats injected with the non-immune IgG-dexamethasone conjugate (Figure 2D).

Ex vivo staining of isolated Kupffer cells incubated with the anti-CD163-dexamethasone conjugate for 30 min demonstrated vesicular uptake (in accordance with receptor-mediated endocytosis) and, to a large extent, co-location of the anti-CD163-antibody and dexamethasone after 30 min of incubation (Figures 3A–3C). In contrast, injection of IgG-dexamethasone conjugate did not lead to staining of isolated Kupffer cells (Figures 3D–3F).

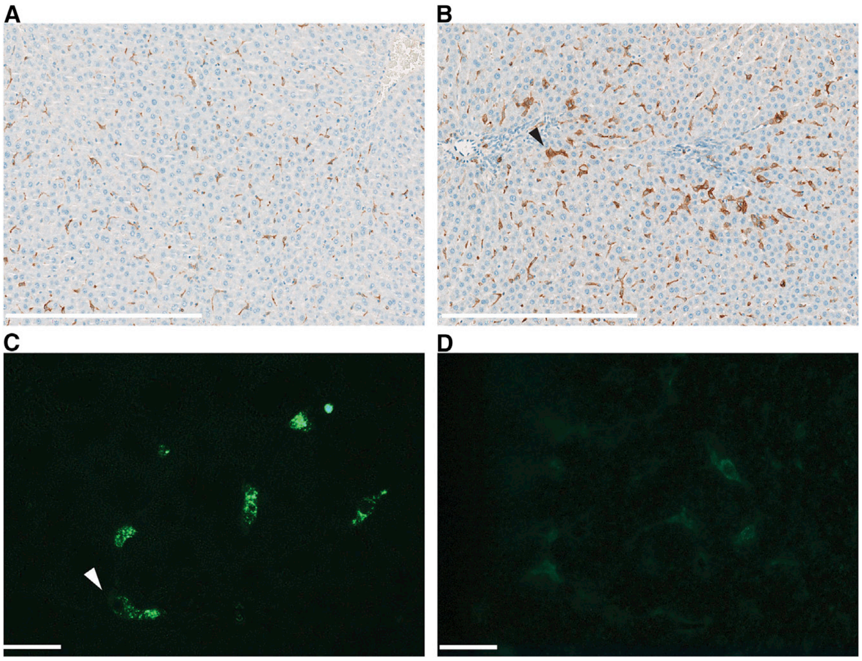

Figure 2. CD163-Positive Liver Macrophages in Rats on the Standard Diet versus the HFr Diet

(A and B) Anti-CD163 antibody staining of Kupffer cells/infiltrating macrophages using immunohistochemistry of fixed paraffin-embedded liver sections from rats fed the (A) STD or (B) HFr diet for 2 weeks. White scale bars represent 400 μm. (C and D) Fluorescence microscopic analysis of formalin-fixed cryosections of rat liver 1 hr after i.v. injection of (C) the anti-CD163-dexamethasone conjugate and (D) the control IgG-dexamethasone conjugate in rats on the STD. Anti-mouse IgG-Alexa Fluor 488 (green) was used for detection of the conjugates. Staining of the CD163-dexamethasone conjugate is in accordance with uptake in Kupffer cells (arrowheads). Scale bars represent 20 μm.

Effect on HFr Diet-Induced Liver Changes by Targeting a Low-Dose GC to CD163-Positive Macrophages

The treatment efficacy of CD163-targeted low-dose dexamethasone was subsequently studied in rats during early NASH disease progression (5 weeks on the HFr diet). Upon 2 weeks on the HFr diet, 32 rats were randomized into four treatment groups (n = 8), maintained on the HFr diet, and treated the following 3 weeks with i.v. injections (three times per week) of vehicle, non-conjugated dexamethasone (0.02 mg/kg), anti-CD163-dexamethasone conjugate (0.02 mg dexamethasone/kg), or control IgG-dexamethasone conjugate (0.02 mg dexamethasone/kg). The treatment groups and doses used in the present study were chosen on the basis of a short endotoxin-induced inflammation experiment in rats (Table S2) with serum TNF-α as the readout, as previously described.[27] The dose response showed the maximal effect of a single preventive injection of the anti-CD163-dexamethasone conjugate at a dose of 0.02 mg dexamethasone/kg. No effect of the same dose of dexamethasone in a free form or conjugated to an irrelevant IgG was seen, nor was there any effect of free anti-CD163 IgG (Table S2) in accordance with fast removal of the antibody by CD163.[27,45]

Three days after the last injection on day 36, the experiment was terminated and the liver status of the rats was evaluated. The group treated with the anti-CD163-dexamethasone conjugate showed strongly reduced values of all analyzed histological liver lesion parameters (steatosis, inflammation, hepatocyte ballooning, fibrosis, and glycogen accumulation) contributing to NASH, relative to the placebo control; accordingly, the NAFLD activity score (NAS) (steatosis, hepatocyte ballooning, and inflammation) was significantly reduced, whereas no effect was seen in rats treated with free dexamethasone (Figure 4; Table 2). Some reduction in the overall score was seen in the group treated with the IgG-dexamethasone conjugate, but steatosis and hepatocyte ballooning contributing to the NAS was significantly exacerbated in the IgG-dexamethasone group compared to the anti-CD163-dexamethasone group ($p = 0.040$ and $p = 0.004$, respectively). Interestingly, Oil Red O staining (Figure 5) showed that rats treated with free dexamethasone or IgG-dexamethasone exhibited more pronounced macrovesicular steatosis, which might be accounted for by a direct lipogenic effect of dexamethasone taken up in hepatocytes. Furthermore, hepatic fibrosis, scored as the sum of pericellular and perivenular fibrosis as well as glycogen deposition, was significantly reduced only in the anti-CD163-dexamethasone group (Figure 4; Table 2).

Figures 6A and 6B illustrate the far higher efficacy of the anti-CD163-dexamethasone conjugate on liver weight and total NASH score (representing the sum of all analyzed histological parameters shown in Table 2), respectively, compared to similar doses of free or IgG-coupled dexamethasone. This significant treatment effect on NASH progression was confirmed by histological analyses in an identical treatment setup including eight rats in each treatment group.

Activity levels of the liver enzymes alanine transaminase (ALT), AST, and lactate dehydrogenase (LDH) were lower in all treatment groups, but the effect on ALT and AST levels was more pronounced in the conjugate treatment groups (Table 2). Levels of glucose, triglyceride, and cholesterol were significantly lower in the anti-CD163-dexamethasone group compared to the IgG-dexamethasone group (Table 2). Serum cytokine TNF-α levels were significantly lower in the conjugate treatment groups compared to the vehicle group, whereas IL-1β serum levels were significantly lowered in all three treatment groups (Table 2).

RNA profiling of selected liver genes of potential relevance to NASH pathology are shown in Table S4, showing that hepatic genes were significantly up- or downregulated more than 1.4-fold, relative to

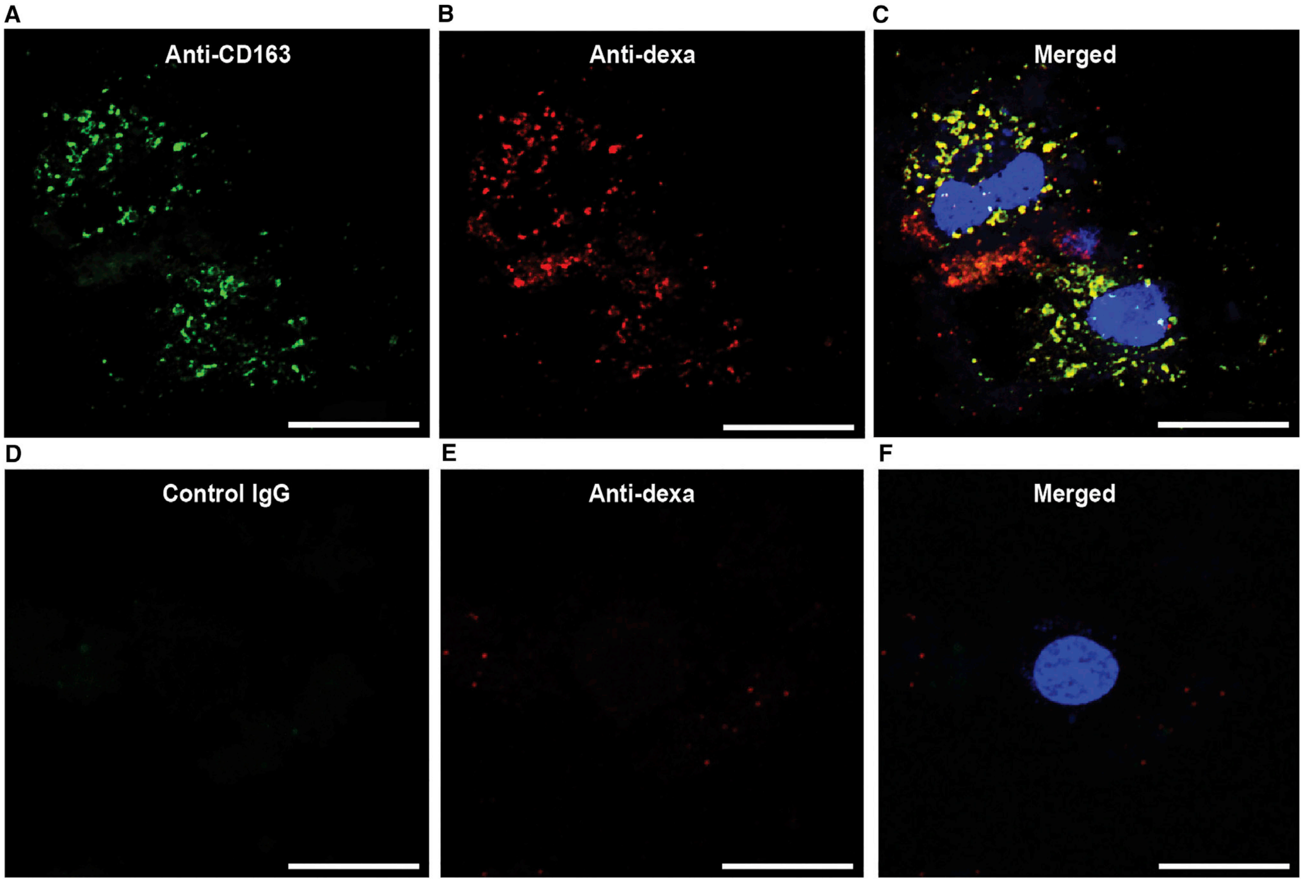

Figure 3. Targeting of Anti-CD163-Dexamethasone to Rat Kupffer Cells

Confocal fluorescence microscopy analysis of liver cell suspensions after 30 min of incubation with (A–C) the anti-CD163-dexamethasone conjugate or (D–F) the IgG-dexamethasone conjugate. (A and D) Anti-mouse IgG-Alexa Fluor 488 (green) staining of CD163 antibody. (B and E) Anti-rabbit Alexa Fluor 647 (red) staining of dexamethasone. (C) shows the overlay of (A) and (B) and (F) shows the overlay of (D) and (E), with DAPI (light blue) nuclei staining showing co-location of anti-CD163 and dexamethasone. Scale bars represent 20 μm.

the vehicle-treated group. Treatment with dexamethasone (free or conjugated) counter-regulated to some extent genes that were up- or downregulated by the HFr diet, but only modest differences were generally seen in the expression patterns of these genes, which are active mainly in hepatocytes. However, of note, the genes involved in mitochondrial β-oxidation were collectively upregulated only in the anti-CD163-dexamethasone group. Also, the interferon-γ gene (*Ifng*), which has counteracting immunoregulatory properties,[46,47] showed a marked upregulation in the anti-CD163-dexamethasone group.

Systemic Effects of the Anti-CD163-Dexamethasone Conjugate

No behavioral changes were observed in the treatment groups. Thymus weight, which is a common measure in rats for the systemic effect of dexamethasone owing to a strong apoptosis-inducing effect on lymphocytes, was significantly lower in the free dexamethasone group compared to vehicle, whereas no effect on thymus size was seen in the conjugate groups (Table 2).

DISCUSSION

The present rat data show a strong positive effect of specific dexamethasone targeting to the M2 macrophage receptor CD163 in an inflammatory model, where fructose induces rapidly progressing steatosis followed by inflammation, hepatocyte ballooning, and fibrosis mimicking human NASH. Targeting Kupffer cells with low-dose anti-CD163 conjugated dexamethasone substantially reduced all histological parameters defining NASH. Furthermore, the fructose-induced increase in liver weight, as well as the development of pericellular and perivenular fibrosis, was prevented and blood parameters of liver inflammation (ALT, AST, triglyceride, TNF-α, and IL-1β) were also reduced. No or little effect on fructose-induced NASH was seen with a similar dose of free dexamethasone. These findings confirm the pivotal role of macrophages in driving the liver inflammation induced by fructose, keeping in mind that the activation of Kupffer cells and macrophage accumulation is a second line of the pathogenesis. Any therapeutic effect of macrophage-directed activity on hepatocytes may only occur indirectly via signaling by

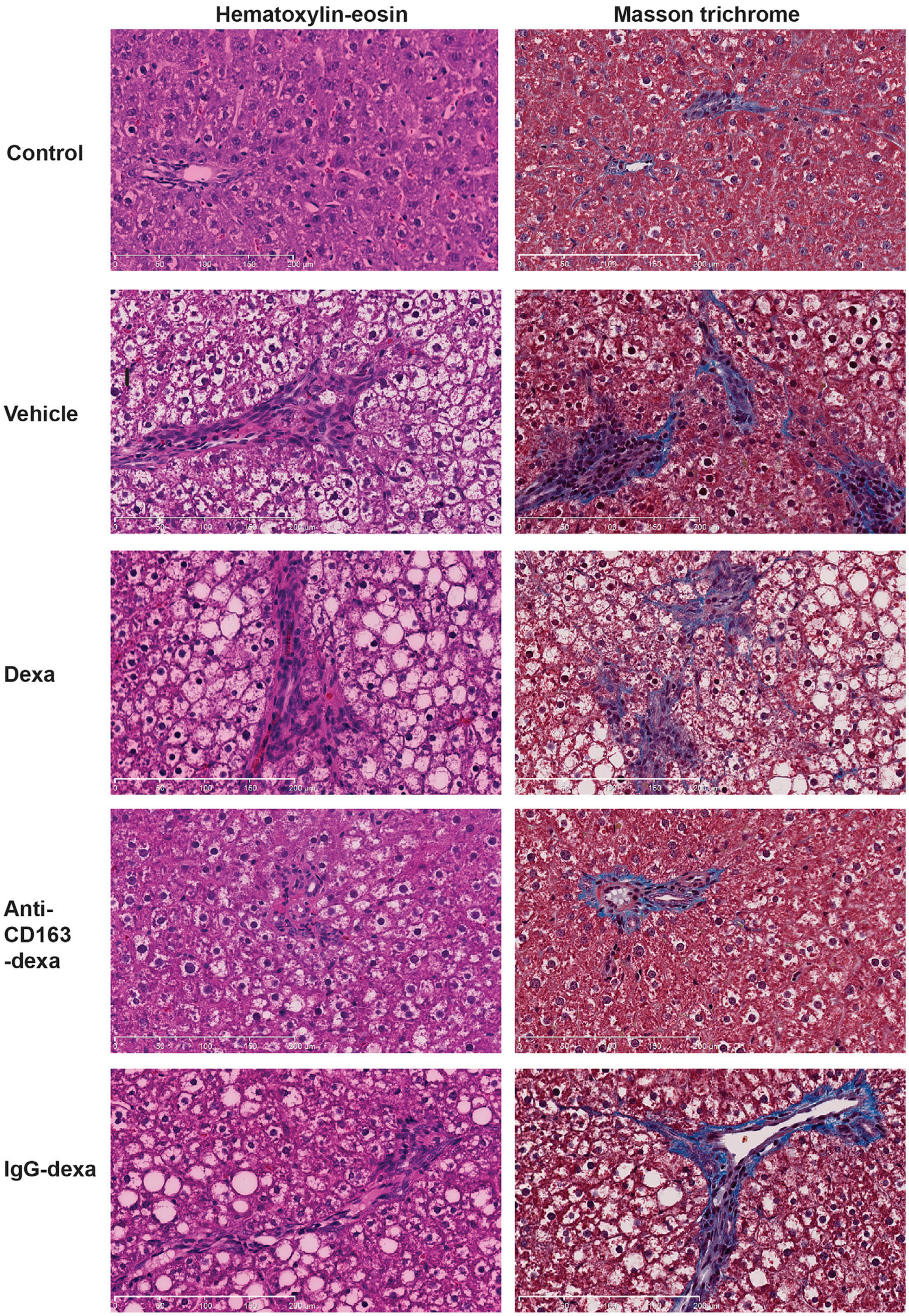

(legend on next page)

Table 2. Organ Weight, Histological Scoring, and Paraclinical Values in Rats Treated with Vehicle, Free Dexamethasone, Anti-CD163-Dexamethasone, and IgG-Dexamethasone

Parameter	Vehicle	Dexa	Anti-CD163-Dexa	IgG-Dexa
Body weight (g)	314.5 ± 3.7	298.1 ± 5.2*	300.7 ± 3.9*	312.1 ± 5.5
Liver/body weight (g/100 g)	5.3 ± 0.10	5.2 ± 0.20	4.8 ± 0.04**	4.9 ± 0.20
Spleen/body weight (g/100 g)	0.22 ± 0.01	0.21 ± 0.01	0.22 ± 0.01	0.25 ± 0.01*
Thymus/body weight (g/100 g)	0.17 ± 0.01	0.12 ± 0.01*	0.15 ± 0.01	0.17 ± 0.01
Steatosis (0–3)	2.88 ± 0.13	2.50 ± 0.19	1.62 ± 0.18**	2.12 ± 0.12**
Inflammation (0–3)	2.12 ± 0.12	1.69 ± 0.25	1.00 ± 0.19**	1.38 ± 0.12**
Hepatocyte ballooning (0–2)	1.67 ± 0.13	1.38 ± 0.10	0.71 ± 0.10**	1.21 ± 0.11*
NAS score (0–8)	6.67 ± 0.28	5.56 ± 0.41	3.33 ± 0.03**	4.71 ± 0.31**
Fibrosis (0–8)	3.75 ± 0.45	3.75 ± 0.50	1.75 ± 0.25**	2.81 ± 0.29
Pericellular (0–4)	1.70 ± 0.34	1.88 ± 0.27	0.75 ± 0.25*	1.19 ± 0.13
Perivenular (0–4)	2.00 ± 0.27	1.88 ± 0.64	1.00 ± 0.19**	1.63 ± 0.18
Glycogen deposition (0–4)	2.19 ± 0.19	1.69 ± 0.25	1.06 ± 0.18**	1.75 ± 0.27
ALT (U/L)	334.1 ± 33.1	192.5 ± 16.5**	158.6 ± 13.7**	128.3 ± 8.5**
AST (U/L)	119.9 ± 19.9	101.4 ± 16.1	64.3 ± 7.6*	68.6 ± 5.3*
LDH (U/L)	2,414 ± 187.7	1,351 ± 102.8**	1,370 ± 75.7**	1,159 ± 124.5**
ALP (mM)	183.5 ± 7.1	198.6 ± 9.0	169.4 ± 6.8	166.6 ± 5.8
Glucose (mM)	9.1 ± 0.8	10.1 ± 0.8	11.5 ± 0.9	13.4 ± 1.3**
Cholesterol (mM)	1.8 ± 0.1	2.1 ± 0.1*	2.1 ± 0.1*	2.8 ± 0.2**
HDL (mM)	0.8 ± 0.1	1.24 ± 0.1**	1.3 ± 0.1**	1.3 ± 0.2
Triglyceride (mM)	3.9 ± 0.3	3.0 ± 0.2*	2.5 ± 0.2**	4.3 ± 1.0
Carbamide (mM)	9.3 ± 0.6	9.1 ± 0.5	6.7 ± 0.4**	7.7 ± 0.6*
Creatinine (mM)	19.2 ± 0.7	17.9 ± 1.5	16.0 ± 0.7*	15.1 ± 0.8**
TNF-α (pg/mL)	7.9 ± 1.1	6.1 ± 0.8	4.6 ± 0.6*	3.6 ± 0.6**
IL-1β (pg/mL)	253.0 ± 71.8	90.1 ± 27.0*	90.1 ± 37.1*	76.3 ± 20.8*

Values are means ± SEM (n = 8 per group). Significant differences between the vehicle group and the dexa, anti-CD163-dexa, and IgG-dexa groups are indicated by *p < 0.05 and **p < 0.01, respectively. dexa, dexamethasone.

macrophages, and this type of therapy may not abolish the primary pathologic mechanisms initiating disease. This is also in accordance with the present data showing that the main effect of the CD163-directed therapy was on the secondary liver changes, whereas less reduction of the direct fructose-induced hepatocyte changes such as steatosis was seen.

Targeting GCs to CD163-positive macrophages has two major advantages. First, it decreases the effective anti-inflammatory dose response approximately 50-fold, as estimated by measuring the cytokine response in rats and pigs after endotoxin injections.[27,29] Second, it reduces off-targeting, which is essential in the liver because of the strong diabetic effects of GCs in hepatocytes. In fact, the present study shows that rats receiving low-dose dexamethasone in non-conjugated form actually developed increased macrovesicular steatosis compared to the non-treated rats. Off-targeting effects in extrahepatic tissues are also a serious therapeutic problem with GCs. In this study, the systemic effect was analyzed by weighing the thymus, which is extremely sensitive to GC-induced apoptosis of its lymphocyte population. Reduced thymus weight in rats treated with free dexamethasone is in line with previous data showing side effects of equal and higher concentrations of free dexamethasone in rats.[27] For comparison, dexamethasone is administered at doses of 1–10 mg per day in the clinic; even without correction by allometric scaling, this dose is substantially higher than the dose used in rats in the present study (0.02 mg dexamethasone/kg, three times per week).

The molecular mechanisms of the NASH-attenuating effect by anti-CD163-dexa treatment may be multiple and need further exploration.

Figure 4. Anti-CD163-Dexamethasone Treatment Strongly Reduces Development of Steatohepatitis and Fibrosis in Rats on the HFr Diet
Treatment effects of free dexamethasone, anti-CD163-dexamethasone, and IgG-dexamethasone conjugates. The left panel represents H&E staining showing fat depositions, inflammation, and hepatocyte ballooning. The right panel represents Masson's trichrome-stained sections showing hepatic regions with fibrosis (blue staining represents collagen deposition). Magnification, ×20. Scale bars represent 200 μm.

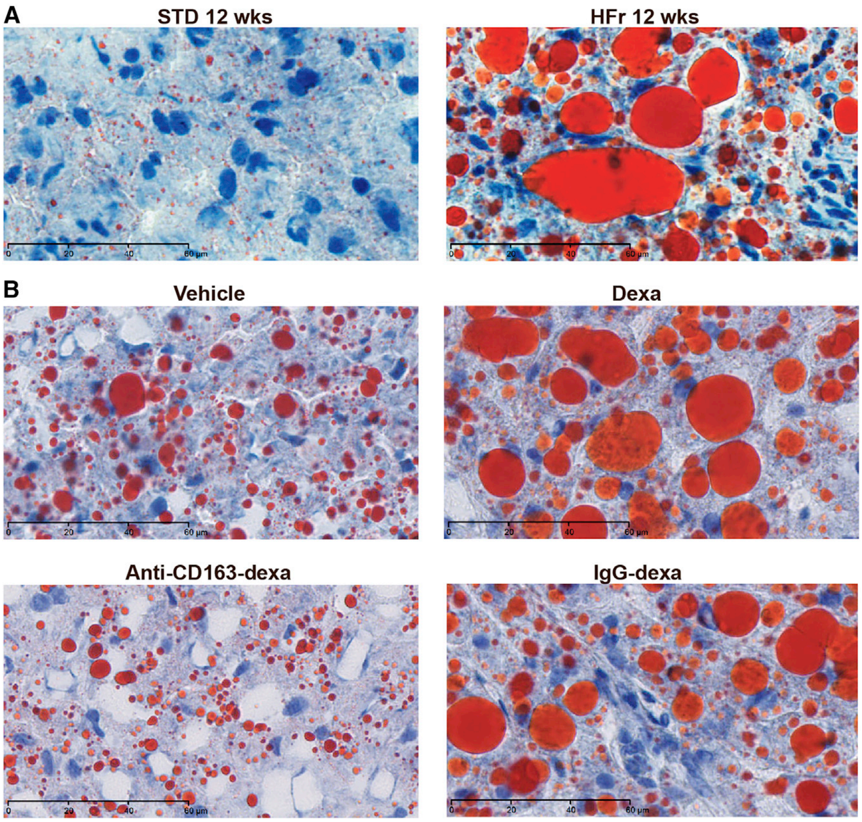

Figure 5. Hepatic Lipid Accumulation Visualized by Red Oil O Staining

Red oil O staining of frozen liver sections counterstained with hematoxylin showing red-colored micro- and macrovesicular fatty droplets in areas of steatosis. The top panel in (A) shows stainings of the liver of a rat on the STD and at 12 weeks on the HFr diet, for comparison with the four treatment groups in (B). Magnification, ×63. Scale bars represent 60 μm.

Previous data have shown that anti-CD163-dexa therapy strongly reduces the acute pro-inflammatory macrophage cytokine storm (TNF-α in particular) induced by LPS[27] that is suggested to play role in NASH development.[47] However, anti-TNF-α therapy has not proved effective in treating NASH in humans,[25] suggesting that other factors are important in disease progression. This is also indicated from results of the present study, where the increase in fructose-induced TNF-α measured at the termination of the study was only partially reduced by anti-C163-dexa, and the effect was not different from that of Ig-CD163-dexa. The transcription level of INF-γ in the anti-C163-dexa-treated animals was also surprising because INF-γ is normally regarded as a pro-inflammatory cytokine. However, IFN-γ has several immunoregulatory properties. IFN-γ has also been shown to specifically inhibit extracellular matrix synthesis, which leads to deposition of scar or fibrous tissue.[48] Further, in vitro studies of the effect of IFN-γ on hepatic stellate cells, which are the most important fibrogenic cells in the liver, demonstrated that this cytokine inhibits multiple aspects of stellate cell activation.[49] Clinical studies with IFN-γ have demonstrated reduction of fibrosis as well as histological reversal of cirrhosis.[46,50] Suppression of collagen1a1 (*Col1a1*) gene expression seems to be an important component in this clinical effect of IFN-γ.[51] In line with these findings, there was a trend that expression of *Col1a1* was specifically decreased by the anti-CD163-dexamethasone conjugate (1.6-fold downregulation, p = 0.07, data not shown).

The Kupffer cell population is the major body macrophage pool, which, in NASH, is further supplemented with infiltrating CD163 macrophages (Figure 2). Accordingly, the majority of injected anti-CD163 antibody is taken up in the liver.[45] A minor, but still a substantial, fraction of injected anti-CD163-dexamethasone may target other tissue macrophages though. How this uptake impacts on NASH is unknown, but along this line macrophages in the adipose tissue are particular interesting because the macrophage M1/M2 balance influences on the lipid metabolism in the adipose tissue and on triglyceride release to plasma.[52] Our data on liver did not indicate a major change in the M1/M2 balance but it is possible that the targeting of dexamethasone to CD163-positive M2 macrophages in the adipose tissue has an indirect regulating effect on altered lipid metabolism in this tissue.

In the liver, several parameters indicated changes in the lipid metabolism. For instance, the transcription of the three genes *Acadl*, *Cpt1a*, and *Fabp1* significantly downregulated in the fructose-induced NASH model, were collectively upregulated in the group treated with anti-CD163-dexamethasone (Table S4). The encoded proteins are highly involved in transport and mitochondrial β-oxidation of fatty acids.[53] Mitochondrial dysfunction caused by impaired β-oxidation may lead to increased production of reactive oxygen species (ROS) that may stimulate pro-inflammatory processes and contribute to insulin resistance and metabolic abnormalities.[53] Fatty acid oxidation has been shown to attenuate inflammatory and endoplasmic reticulum stress responses in human macrophages.[54,55] Thus, the anti-inflammatory potency and NASH-protective effect of the anti-CD163-dexamethasone conjugate in rats on HFr diet might to some extent relate to increased β-oxidation of fatty acids originating from the conversion of fructose to triglycerides.

Although it was significantly less effective than anti-CD163 dexamethasone, the IgG-dexamethasone conjugate also had some reducing effect on NASH progression. The IgG-dexamethasone conjugate was shown to distribute differently in the liver and showed no binding or uptake in isolated Kupffer cells. Due to the long circulation

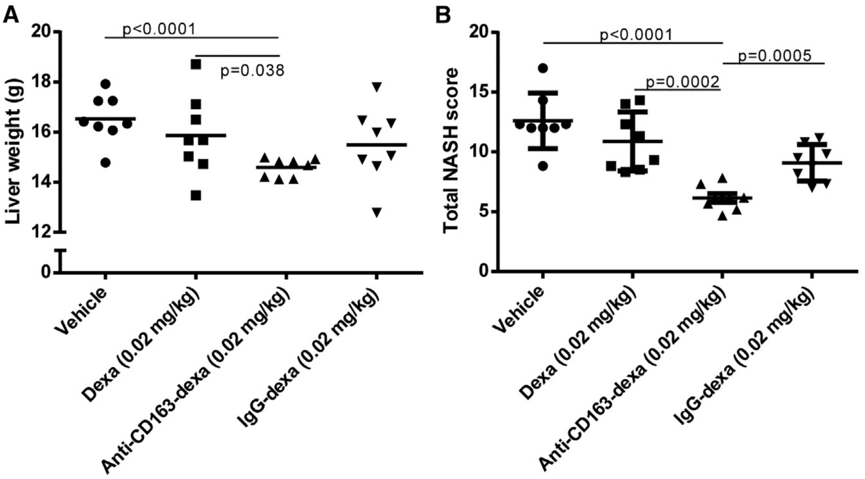

Figure 6. Liver Weight and Total NASH Score in Treatment Groups of Rats with HFr-Induced NASH

(A) Treatment effect on liver weight in different treatment groups (n = 8) of rats on the HFr diet. (B) Treatment effects on total NASH score, defined as the sum of steatosis (0–3), inflammation (0–3), hepatocyte ballooning (0–2), fibrosis (0–8), and glycogen deposition (0–4). Lines represent means ± SEM.

time of IgG, dexamethasone bound to non-immune IgG is likely to be released in plasma, and perhaps it also to some extent targets Fc receptors on monocytic cells and other immune cells. However, increased levels of serum TG and glucose compared to the vehicle group indicate metabolic side effects of this non-CD163-targeted conjugate. In line with these observations, RNA profiling showed significant upregulation of genes involved in fatty acid metabolism and glucose uptake and transport in rats treated with IgG-dexamethasone.

Targeting of dexamethasone to macrophages has also been investigated by others[56] using macrophage-targeting mannosylated albumin with linked dexamethasone in a rat model of liver fibrosis induced by bile duct ligation. In line with our data, a decreased TNF-α response and reactive oxygen species generation was seen, indicating less inflammatory response. However, in stark contrast with our data, no attenuation of fibrosis was observed in that model. The model based on bile intoxication might stimulate the fibrosis-stimulating stellate cells,[57] which could be one reason for this difference. Furthermore, the mannose receptor used for targeting is also expressed in endothelial cells.[58]

In conclusion, selective anti-CD163-dexamethasone targeting of Kupffer cells with a low-dose dexamethasone conjugate prevented development of fructose-induced steatohepatitis in rats without apparent serious systemic side effects. The data therefore point to the CD163-positive macrophage population as a potential therapeutic target to prevent progression of further liver damage in patients with NASH with increased caloric intake, although such medical therapy may not substitute dietary interventions. Furthermore, the macrophage-targeting principle with GCs or other anti-inflammatory drugs might also be relevant for other inflammatory diseases, including other liver diseases such as acute alcoholic hepatitis, which shares pathology with NASH and has a several-fold accumulation of CD163-positive macrophages in the liver.[59,60] This disease has high mortality and there is an imminent need for new targeted approaches.[26]

Dexamethasone was the GC chosen in the present anti-CD163-antibody conjugate. Dexamethasone is a widely used high-potency GC with no or low mineralocorticoid activity. Selective macrophage targeting of GCs may, to a large extent, circumvent the problem of systemic site effects, mainly because of a much lower effective dose of GCs. As an alternative approach to develop safer GCs, a range of synthetic GCs with an apparent more selective action on the GC receptor have been developed by others with the aim to promote a stronger anti-inflammatory profile.[61] So far, such new GC compounds have not proved successful in clinical studies, perhaps (most likely) because activation of the glucocorticoid receptor inevitably leads to the pleiotropic effects of GCs. However, it seems likely that further optimization of GC therapy may be achieved by combining chemical GC development and targeting to macrophages.

MATERIALS AND METHODS

Conjugate Preparations

The IgG-dexamethasone conjugate was synthesized by conjugating dexamethasone (on average, four drug molecules per IgG) to primary amino groups of either the murine anti-rat CD163 antibody (ED2; Bio-Rad AbD Serotec) or to murine IgG (Sigma-Aldrich) as described previously using a hemisuccinate linker.[27]

Fluorescence Microscopy

Male Sprague-Dawley rats (Taconic) were injected (i.v.) with anti-CD163-dexamethasone (0.02 mg/kg) (n = 2) or IgG-dexamethasone (0.02 mg/kg) (n = 2). One hour after injections, the rats were anesthetized and perfused with ice-cold PBS before the livers were trimmed into small blocks and fixed by immersion fixation with 10% neutral buffered formalin solution (Sigma-Aldrich) for 2 hr. The livers were further fixed for 24 hr in 1% neutral buffered formalin, infiltrated with 2.3 M sucrose for 30 min, and then frozen in liquid nitrogen. One-micrometer cryosections for light microscopy (LM) were obtained with a Leica EM FC6 cryo-ultramicrotome (Leica Microsystems). For immunofluorescence, the sections were preincubated in PBS containing 0.02 M glycine and 0.5% bovine serum albumin followed by incubation for 1 hr with goat anti-mouse IgG-Alexa Fluor 488 antibody (Abcam) for detection of the anti-CD163 antibody and the control IgG. The stained sections were mounted with hydrophilic mounting media containing an anti-fading reagent (DAKO) and were examined by a Leica DMR microscope equipped with a Leica DFC320 camera (Leica Microsystems).

Further characterization of hepatic binding of the anti-CD163-dexamethasone conjugate was performed ex vivo by analysis of liver cell suspensions. Small pieces of rat liver were digested using collagenase VI (2 mg/g tissue; Sigma-Aldrich) at 37°C for 30 min in Hank's balanced salt solution with calcium and magnesium chloride (Life Technologies) and 2% BSA before they were filtered through a cell strainer and washed by centrifugation. The cell suspensions were seeded in DMEM supplemented with 10% FBS in chamber slides for 2 hr before gentle washing of the attached liver cells and further incubation overnight. The cells were rinsed and incubated with either the anti-CD163-dexamethasone or murine IgG-dexamethasone conjugate for 30 min. Dexamethasone was detected with a rabbit anti-dexamethasone antibody (Abcam). A goat anti-rabbit Alexa Fluor 647 antibody (Abcam) and a goat anti-mouse IgG-Alexa Fluor 488 antibody (Abcam) were used as secondary antibodies for staining of the conjugates. After fixation of the cells, the chamber slides were covered with mounting medium containing DAPI (Life Technologies) before laser confocal fluorescence microscopy for visualization of stained cells using a Zeiss LSM-710 confocal microscope system (Carl Zeiss MicroImaging).

Rat Model and Dexamethasone Treatment

The experimental protocol was approved by the Danish Experimental Animal Inspectorate (permission no. 2012-15-2934-00250). Male Sprague-Dawley rats (120–140 g) were purchased from Taconic. The animals were maintained under controlled temperature (20 ± 2°C) and light (lights on 08:00–20:00 hr) and fed a standard diet (Altromin) for 7 days before they were randomly separated into diet or treatment groups. All rats had free access to the respective diet and water during the experimental periods. High fructose (70% fructose, 10% fat, and 20% protein) was purchased from Research Diet.

In the initial time study to validate the model, the control group was on the STD diet, while the HFr group received the high-fructose diet specified above. Once a week for 12 weeks, four animals from the HFr group were randomly selected for analysis of biochemical blood parameters, liver histology, and RNA profiling. The animals were fasted for 20 hr before termination. The final group fed the HFr diet was analyzed after 12 weeks (n = 8) and the control group on the STD diet was analyzed after 2 weeks (n = 4) and 12 weeks (n = 8). At termination, the rats were anesthetized with isoflurane and the abdomen was opened. Following blood collection from the left ventricle, the rats were perfused with ice-cold PBS before dissection of small tissue pieces from the left lobe of the liver for RNA preparation. Subsequently, the rats were perfused with 10% neutral buffered formalin and organs were dissected and further fixed in 10% neutral buffered formalin for 24 hr before the determination of organ weights and the isolation of liver portions from the left lobe.

In the treatment experiment that was performed twice, the rats were fed the HFr diet for 2 weeks before being randomly divided into four treatment groups (n = 8) and continued on the HFr diet. The rats were injected (i.v.) three times per week for 3 weeks with vehicle (PBS), dexamethasone-phosphate (0.02 mg/kg; Sigma), anti-CD163-dexamethasone (0.02 mg/kg), or mouse IgG-dexamethasone (0.02 mg/kg). Body weight and the amount of food and water intake were determined three times a week. We found no significant differences between consumption (weight) of food among the groups (Table S3). The experiment was terminated 3 days after the ninth injection, as described above.

Liver Histology and Immunohistochemistry

Histological assessment of the liver conditions was analyzed using H&E, Masson's trichrome, and periodic acid-Schiff stains of 3-μm formalin-fixed paraffin-embedded sections. The disease severity was blindly evaluated according to the NAFLD scoring system.[62] The NAS, ranging from 0 to 8, was determined from the degree of steatosis (0–3), hepatocyte ballooning (0–2), and lobular inflammation (0–3). According to the NASH Clinical Research Network, a NAS of ≥ 5 corresponds to a diagnosis of definitive NASH. Fibrosis severity was scored 0–8 and defined by the sum of pericellular (0–4) and perivenular (0–4) fibrosis scores (modified from Kleiner et al.[62]). Glycogen deposition was scored from 0 to 4. Paraffin-embedded liver tissue sections (3 μm) were analyzed by immunochemistry using a mouse monoclonal anti-rat CD163 antibody (ED2; Bio-Rad AbD Serotec) for detection of CD163 and visualized by peroxidase conjugated secondary antibody. Lipid and fat accumulation was analyzed by Oil Red O staining of cryosections from frozen liver tissue. Quantitative measurements of staining were determined using ImageJ software (NIH).

Measurement of Paraclinical Parameters

Blood samples were processed to serum and stored at −80°C. A Roche Hitachi Cobas 6000 (Roche Diagnostics) analyzer was used to measure serum activity of ALT, AST, LDH, and alkaline phosphatase (ALP), as well as concentrations of glucose, triglyceride, total cholesterol, and high-density lipoprotein (HDL) cholesterol. Serum levels of IL-1β and TNF-α were determined using commercial ELISAs (Life Technologies) according to the manufacturer's recommendations.

Statistical Analyses

All data variables were tested for normal distribution. Normally distributed variables are presented as means ± SEM. Multiple rat groups were analyzed and compared for treatment effects using two-way ANOVA. For comparison of the two groups, normally distributed data were tested with the Student's t test. Nonparametric data were tested using the Kruskal-Wallis test, followed by the Wilcoxon Mann-Whitney rank sum test. p values < 0.05 were considered significant. All statistical analyses were performed using GraphPad Prism software (version 6.0 for Windows).

SUPPLEMENTAL INFORMATION

Supplemental Information includes Supplemental Materials and Methods, two figures, and five tables and can be found with this article online at http://dx.doi.org/10.1016/j.omtm.2016.11.004.

AUTHOR CONTRIBUTIONS

P.S. performed the animal experiments and data analysis and contributed to study design and writing, J.H.G. made the antibody-drug conjugates. A.E. contributed to the gene analyses; H.H., R.R., and E.I.C. performed the pathological and microscopic analyses; H.J.M. performed biochemical analyses; H.G. and H.V. advised; and S.K.M. supervised and contributed to study design and writing.

CONFLICTS OF INTEREST

J.H.G., H.J.M., and S.K.M. are minority shareholders in Affinicon ApS.

ACKNOWLEDGMENTS

We thank Zane Binate for technical assistance. This work was supported by the European Research Council (ERC) (TROJA program advanced grant 233312 and NASH program PoC grant 324613), the Novo Nordisk Foundation, the Danish Medical Research Council, and the Danish Strategic Council.

REFERENCES

1. Woo Baidal, J.A., and Lavine, J.E. (2016). The intersection of nonalcoholic fatty liver disease and obesity. Sci. Trans. Med. *8*, 323rv1.
2. Fan, J.G., and Cao, H.X. (2013). Role of diet and nutritional management in non-alcoholic fatty liver disease. J. Gastroenterol. Hepatol. *28* (*Suppl 4*), 81–87.
3. Bray, G.A. (2010). Soft drink consumption and obesity: it is all about fructose. Curr. Opin. Lipidol. *21*, 51–57.
4. Sanyal, A.J., Friedman, S.L., McCullough, A.J., and Dimick-Santos, L.; American Association for the Study of Liver Diseases; United States Food and Drug Administration (2015). Challenges and opportunities in drug and biomarker development for nonalcoholic steatohepatitis: findings and recommendations from an American Association for the Study of Liver Diseases-U.S. Food and Drug Administration Joint Workshop. Hepatology *61*, 1392–1405.
5. Wong, R.J., Aguilar, M., Cheung, R., Perumpail, R.B., Harrison, S.A., Younossi, Z.M., and Ahmed, A. (2015). Nonalcoholic steatohepatitis is the second leading etiology of liver disease among adults awaiting liver transplantation in the United States. Gastroenterology *148*, 547–555.
6. Bettermann, K., Hohensee, T., and Haybaeck, J. (2014). Steatosis and steatohepatitis: complex disorders. Int. J. Mol. Sci. *15*, 9924–9944.
7. Duwaerts, C.C., and Maher, J.J. (2014). Mechanisms of liver injury in non-alcoholic steatohepatitis. Curr. Hepatol. Rep. *13*, 119–129.
8. Kawasaki, T., Igarashi, K., Koeda, T., Sugimoto, K., Nakagawa, K., Hayashi, S., Yamaji, R., Inui, H., Fukusato, T., and Yamanouchi, T. (2009). Rats fed fructose-enriched diets have characteristics of nonalcoholic hepatic steatosis. J. Nutr. *139*, 2067–2071.
9. Ackerman, Z., Oron-Herman, M., Grozovski, M., Rosenthal, T., Pappo, O., Link, G., and Sela, B.A. (2005). Fructose-induced fatty liver disease: hepatic effects of blood pressure and plasma triglyceride reduction. Hypertension *45*, 1012–1018.
10. Kohli, R., Kirby, M., Xanthakos, S.A., Softic, S., Feldstein, A.E., Saxena, V., Tang, P.H., Miles, L., Miles, M.V., Balistreri, W.F., et al. (2010). High-fructose, medium chain trans fat diet induces liver fibrosis and elevates plasma coenzyme Q9 in a novel murine model of obesity and nonalcoholic steatohepatitis. Hepatology *52*, 934–944.
11. Tilg, H., and Moschen, A.R. (2010). Evolution of inflammation in nonalcoholic fatty liver disease: the multiple parallel hits hypothesis. Hepatology *52*, 1836–1846.
12. Schuppan, D., and Schattenberg, J.M. (2013). Non-alcoholic steatohepatitis: pathogenesis and novel therapeutic approaches. J. Gastroenterol. Hepatol. *28* (*Suppl 1*), 68–76.
13. Rivera, C.A., Adegboyega, P., van Rooijen, N., Tagalicud, A., Allman, M., and Wallace, M. (2007). Toll-like receptor-4 signaling and Kupffer cells play pivotal roles in the pathogenesis of non-alcoholic steatohepatitis. J. Hepatol. *47*, 571–579.
14. Tosello-Trampont, A.C., Landes, S.G., Nguyen, V., Novobrantseva, T.I., and Hahn, Y.S. (2012). Kuppfer cells trigger nonalcoholic steatohepatitis development in diet-induced mouse model through tumor necrosis factor-α production. J. Biol. Chem. *287*, 40161–40172.
15. Vos, M.B., and Lavine, J.E. (2013). Dietary fructose in nonalcoholic fatty liver disease. Hepatology *57*, 2525–2531.
16. Samuel, V.T. (2011). Fructose induced lipogenesis: from sugar to fat to insulin resistance. Trends Endocrinol. Metab. *22*, 60–65.
17. Doycheva, I., and Loomba, R. (2014). Effect of metformin on ballooning degeneration in nonalcoholic steatohepatitis (NASH): when to use metformin in nonalcoholic fatty liver disease (NAFLD). Adv. Ther. *31*, 30–43.
18. Sanyal, A.J., Chalasani, N., Kowdley, K.V., McCullough, A., Diehl, A.M., Bass, N.M., Neuschwander-Tetri, B.A., Lavine, J.E., Tonascia, J., Unalp, A., et al.; NASH CRN (2010). Pioglitazone, vitamin E, or placebo for nonalcoholic steatohepatitis. N. Engl. J. Med. *362*, 1675–1685.
19. Mahady, S.E., Webster, A.C., Walker, S., Sanyal, A., and George, J. (2011). The role of thiazolidinediones in non-alcoholic steatohepatitis - a systematic review and meta analysis. J. Hepatol. *55*, 1383–1390.
20. Armstrong, M.J., Hull, D., Guo, K., Barton, D., Hazlehurst, J.M., Gathercole, L.L., Nasiri, M., Yu, J., Gough, S.C., Newsome, P.N., and Tomlinson, J.W. (2016). Glucagon-like peptide 1 decreases lipotoxicity in non-alcoholic steatohepatitis. J. Hepatol. *64*, 399–408.
21. Carbone, L.J., Angus, P.W., and Yeomans, N.D. (2016). Incretin-based therapies for the treatment of non-alcoholic fatty liver disease: a systematic review and meta-analysis. J. Gastroenterol. Hepatol. *31*, 23–31.
22. Younossi, Z.M., Reyes, M.J., Mishra, A., Mehta, R., and Henry, L. (2014). Systematic review with meta-analysis: non-alcoholic steatohepatitis - a case for personalised treatment based on pathogenic targets. Aliment. Pharmacol. Ther. *39*, 3–14.
23. Ratziu, V. (2013). Pharmacological agents for NASH. Nat. Rev. Gastroenterol. Hepatol. *10*, 676–685.
24. Nanki, T., Koike, R., and Miyasaka, N. (1999). Subacute severe steatohepatitis during prednisolone therapy for systemic lupus erythematosis. Am. J. Gastroenterol. *94*, 3379.
25. Mathurin, P., Louvet, A., Duhamel, A., Nahon, P., Carbonell, N., Boursier, J., Anty, R., Diaz, E., Thabut, D., Moirand, R., et al. (2013). Prednisolone with vs without pentoxifylline and survival of patients with severe alcoholic hepatitis: a randomized clinical trial. JAMA *310*, 1033–1041.
26. Mandrekar, P., Bataller, R., Tsukamoto, H., and Gao, B. (2016). Alcoholic hepatitis: translational approaches to develop targeted therapies. Hepatology *64*, 1343–1355.
27. Graversen, J.H., Svendsen, P., Dagnæs-Hansen, F., Dal, J., Anton, G., Etzerodt, A., Petersen, M.D., Christensen, P.A., Møller, H.J., and Moestrup, S.K. (2012). Targeting the hemoglobin scavenger receptor CD163 in macrophages highly increases the anti-inflammatory potency of dexamethasone. Mol. Ther. *20*, 1550–1558.
28. Etzerodt, A., and Moestrup, S.K. (2013). CD163 and inflammation: biological, diagnostic, and therapeutic aspects. Antioxid. Redox Signal. *18*, 2352–2363.
29. Granfeldt, A., Hvas, C.L., Graversen, J.H., Christensen, P.A., Petersen, M.D., Anton, G., Svendsen, P., Sølling, C., Etzerodt, A., Tønnesen, E., et al. (2013). Targeting dexamethasone to macrophages in a porcine endotoxemic model. Crit. Care Med. *41*, e309–e318.
30. Kristiansen, M., Graversen, J.H., Jacobsen, C., Sonne, O., Hoffman, H.J., Law, S.K., and Moestrup, S.K. (2001). Identification of the haemoglobin scavenger receptor. Nature *409*, 198–201.
31. Andersen, C.B., Torvund-Jensen, M., Nielsen, M.J., de Oliveira, C.L., Hersleth, H.P., Andersen, N.H., Pedersen, J.S., Andersen, G.R., and Moestrup, S.K. (2012). Structure of the haptoglobin-haemoglobin complex. Nature *489*, 456–459.
32. Baek, J.H., D'Agnillo, F., Vallelian, F., Pereira, C.P., Williams, M.C., Jia, Y., Schaer, D.J., and Buehler, P.W. (2012). Hemoglobin-driven pathophysiology is an in vivo consequence of the red blood cell storage lesion that can be attenuated in guinea pigs by haptoglobin therapy. J. Clin. Invest. *122*, 1444–1458.
33. Van Gorp, H., Delputte, P.L., and Nauwynck, H.J. (2010). Scavenger receptor CD163, a Jack-of-all-trades and potential target for cell-directed therapy. Mol. Immunol. *47*, 1650–1660.

34. De Vito, R., Alisi, A., Masotti, A., Ceccarelli, S., Panera, N., Citti, A., Salata, M., Valenti, L., Feldstein, A.E., and Nobili, V. (2012). Markers of activated inflammatory cells correlate with severity of liver damage in children with nonalcoholic fatty liver disease. Int. J. Mol. Med. *30*, 49–56.

35. Younossi, Z.M., Page, S., Rafiq, N., Birerdinc, A., Stepanova, M., Hossain, N., Afendy, A., Younoszai, Z., Goodman, Z., and Baranova, A. (2011). A biomarker panel for non-alcoholic steatohepatitis (NASH) and NASH-related fibrosis. Obes. Surg. *21*, 431–439.

36. Zhang, Y., Baker, S.S., Baker, R.D., Zhu, R., and Zhu, L. (2012). Systematic analysis of the gene expression in the livers of nonalcoholic steatohepatitis: implications on potential biomarkers and molecular pathological mechanism. PLoS ONE *7*, e51131.

37. Pearce, S.G., Thosani, N.C., and Pan, J.J. (2013). Noninvasive biomarkers for the diagnosis of steatohepatitis and advanced fibrosis in NAFLD. Biomark. Res. *1*, 7.

38. Yilmaz, Y. (2013). Biomarkers for early detection of non-alcoholic steatohepatitis: implications for drug development and clinical trials. Curr. Drug Targets *14*, 1357–1366.

39. Joyce, C., Freeman, L., Brewer, H.B., Jr., and Santamarina-Fojo, S. (2003). Study of ABCA1 function in transgenic mice. Arterioscler. Thromb. Vasc. Biol. *23*, 965–971.

40. Musso, G., Cassader, M., Bo, S., De Michieli, F., and Gambino, R. (2013). Sterol regulatory element-binding factor 2 (SREBF-2) predicts 7-year NAFLD incidence and severity of liver disease and lipoprotein and glucose dysmetabolism. Diabetes *62*, 1109–1120.

41. Fon Tacer, K., and Rozman, D. (2011). Nonalcoholic Fatty liver disease: focus on lipoprotein and lipid deregulation. J. Lipids *2011*, 783976.

42. Seo, J.A., Kim, N.H., Park, S.Y., Kim, H.Y., Ryu, O.H., Lee, K.W., Lee, J., Kim, D.L., Choi, K.M., Baik, S.H., et al. (2008). Serum retinol-binding protein 4 levels are elevated in non-alcoholic fatty liver disease. Clin. Endocrinol. (Oxf.) *68*, 555–560.

43. Alkhouri, N., Lopez, R., Berk, M., and Feldstein, A.E. (2009). Serum retinol-binding protein 4 levels in patients with nonalcoholic fatty liver disease. J. Clin. Gastroenterol. *43*, 985–989.

44. Barbé, E., Damoiseaux, J.G., Döpp, E.A., and Dijkstra, C.D. (1990). Characterization and expression of the antigen present on resident rat macrophages recognized by monoclonal antibody ED2. Immunobiology *182*, 88–99.

45. Eichendorff, S., Svendsen, P., Bender, D., Keiding, S., Christensen, E.I., Deleuran, B., and Moestrup, S.K. (2015). Biodistribution and PET imaging of a novel [68Ga]-anti-CD163-antibody conjugate in rats with collagen-induced arthritis and in controls. Mol. Imaging Biol. *17*, 87–93.

46. Gao, B., and Radaeva, S. (2013). Natural killer and natural killer T cells in liver fibrosis. Biochim. Biophys. Acta. *1832*, 1061–1069.

47. Arrese, M., Cabrera, D., Kalergis, A.M., and Feldstein, A.E. (2016). Innate immunity and inflammation in NAFLD/NASH. Dig. Dis. Sci. *61*, 1294–1303.

48. Czaja, M.J., Weiner, F.R., Eghbali, M., Giambrone, M.A., Eghbali, M., and Zern, M.A. (1987). Differential effects of gamma-interferon on collagen and fibronectin gene expression. J. Biol. Chem. *262*, 13348–13351.

49. Gao, B., Radaeva, S., and Jeong, W.I. (2007). Activation of natural killer cells inhibits liver fibrosis: a novel strategy to treat liver fibrosis. Expert Rev. Gastroenterol. Hepatol. *1*, 173–180.

50. Rockey, D.C. (2008). Current and future anti-fibrotic therapies for chronic liver disease. Clin. Liver Dis. *12*, 939–962, xi.

51. Muir, A.J., Sylvestre, P.B., and Rockey, D.C. (2006). Interferon gamma-1b for the treatment of fibrosis in chronic hepatitis C infection. J. Viral Hepat. *13*, 322–328.

52. Schipper, H.S., Prakken, B., Kalkhoven, E., and Boes, M. (2012). Adipose tissue-resident immune cells: key players in immunometabolism. Trends Endocrinol. Metab. *23*, 407–415.

53. Kim, S.Y., Kim, T.B., Moon, K.A., Kim, T.J., Shin, D., Cho, Y.S., Moon, H.B., and Lee, K.Y. (2008). Regulation of pro-inflammatory responses by lipoxygenases via intracellular reactive oxygen species in vitro and in vivo. Exp. Mol. Med. *40*, 461–476.

54. Namgaladze, D., Lips, S., Leiker, T.J., Murphy, R.C., Ekroos, K., Ferreiros, N., Geisslinger, G., and Brüne, B. (2014). Inhibition of macrophage fatty acid β-oxidation exacerbates palmitate-induced inflammatory and endoplasmic reticulum stress responses. Diabetologia *57*, 1067–1077.

55. Namgaladze, D., and Brüne, B. (2016). Macrophage fatty acid oxidation and its roles in macrophage polarization and fatty acid-induced inflammation. Biochim. Biophys. Acta *1861*, 1796–1807.

56. Melgert, B.N., Olinga, P., Van Der Laan, J.M., Weert, B., Cho, J., Schuppan, D., Groothuis, G.M., Meijer, D.K., and Poelstra, K. (2001). Targeting dexamethasone to Kupffer cells: effects on liver inflammation and fibrosis in rats. Hepatology *34*, 719–728.

57. Kim, K.H., Lee, J.M., Zhou, Y., Harpavat, S., and Moore, D.D. (2016). Glucocorticoids have opposing effects on liver fibrosis in hepatic stellate and immune cells. Mol. Endocrinol. *30*, 905–916.

58. Schlesinger, P.H., Doebber, T.W., Mandell, B.F., White, R., DeSchryver, C., Rodman, J.S., Miller, M.J., and Stahl, P. (1978). Plasma clearance of glycoproteins with terminal mannose and N-acetylglucosamine by liver non-parenchymal cells. Studies with beta-glucuronidase, N-acetyl-beta-D-glucosaminidase, ribonuclease B and agalacto-orosomucoid. Biochem. J. *176*, 103–109.

59. Sandahl, T.D., Grønbaek, H., Møller, H.J., Støy, S., Thomsen, K.L., Dige, A.K., Agnholt, J., Hamilton-Dutoit, S., Thiel, S., and Vilstrup, H. (2014). Hepatic macrophage activation and the LPS pathway in patients with alcoholic hepatitis: a prospective cohort study. Am. J. Gastroenterol. *109*, 1749–1756.

60. Szabo, G. (2015). Gut-liver axis in alcoholic liver disease. Gastroenterology *148*, 30–36.

61. Caratti, G., Matthews, L., Poolman, T., Kershaw, S., Baxter, M., and Ray, D. (2015). Glucocorticoid receptor function in health and disease. Clin. Endocrinol. (Oxf.) *83*, 441–448.

62. Kleiner, D.E., Brunt, E.M., Van Natta, M., Behling, C., Contos, M.J., Cummings, O.W., Ferrell, L.D., Liu, Y.C., Torbenson, M.S., Unalp-Arida, A., et al.; Nonalcoholic Steatohepatitis Clinical Research Network (2005). Design and validation of a histological scoring system for nonalcoholic fatty liver disease. Hepatology *41*, 1313–1321.

OMTM, Volume *4*

Supplemental Information

Antibody-Directed Glucocorticoid Targeting to CD163 in M2-type Macrophages Attenuates Fructose-Induced Liver Inflammatory Changes

Pia Svendsen, Jonas H. Graversen, Anders Etzerodt, Henrik Hager, Rasmus Røge, Henning Grønbæk, Erik I. Christensen, Holger J. Møller, Hendrik Vilstrup, and Søren K. Moestrup

Supplementary Material

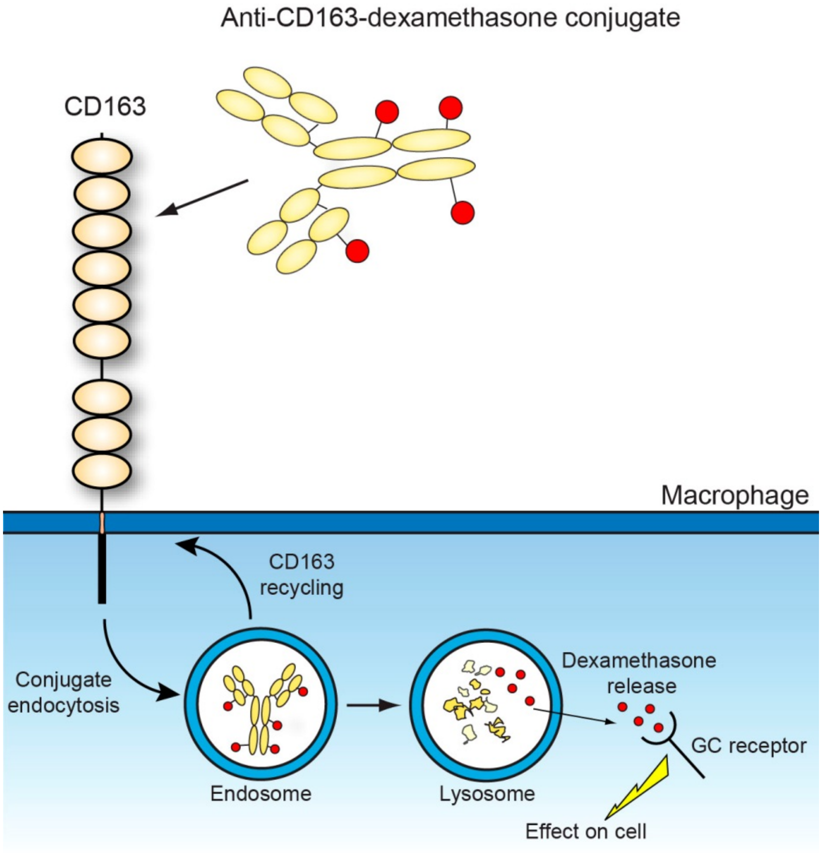

Figure S1. Mechanism of action of anti-CD163 IgG-dexamethasone conjugates. The conjugate that in average contains four dexamethasone drug molecules per IgG protein linked with a breakable ester bonds binds to the haptoglobin-hemoglobin receptor, CD163. This macrophage-specific receptor mediates a fast endocytosis leading to lysosomal degradation of the anti-CD163-dexamethasone conjugate. Dexamethasone now released can diffuse into the cytosol where the drug effect is achieved by binding to the cytosolic GC receptor.

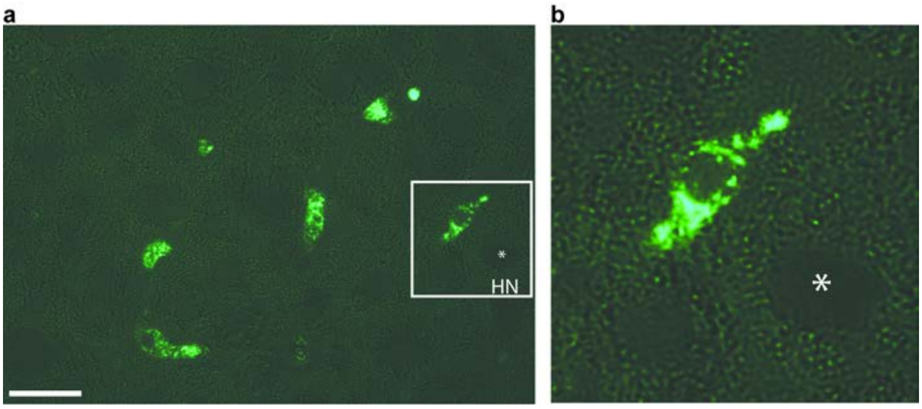

Figure S2. Fluorescence microscopic bright-field analysis of formalin-fixed cryosection of the rat liver one-hour post i.v. injection of the anti-CD163-dexamethasone conjugate. Anti-mouse IgG-Alexa Fluor 488 (green) was used for detection of the conjugate. (b) Enlargement of area indicated in (a) showing vesicular staining in accordance with uptake in Kupffer cell. White scale bar equals 20. *indicates hepatotocyte cell nucleus(HN).

Table S1. Liver mRNA profiling of NASH associated genes significantly up- or down-regulated above a 1.4 fold level in rats feed on HFr diet compared to STD.

Pathway/response	Gene symbol	Encoded protein	Fold Regulation	P-value
**Carbohydrate metabolism**				
	Gk	Glycerol kinase	-2.1	0.005
	G6pd	Glucose-6-phosphatase, catalytic subunit	5.2	0.029
	Pklr	Pyruvate kinase, liver and red blood cell	2.1	0.003
	Rbp4	Retinol binding protein 4, plasma	-1.5	0.005
**Lipid and fatty acid metabolism**				
	Acaca	Acetyl-coenzyme A carboxylase	2.9	0.002
	Acsl5	Acyl-CoA synthetase long-chain family member 5	3.1	0.001
	Acly	ATP citrate lyase	4.9	0.031
	Acsm3	Acyl-CoA synthetase medium-chain family member 3	-4.5	0.006
	Fabp5	Fatty acid binding protein 5, epidermal	3.0	0.031
	Fasn	Fatty acid synthase	6.4	0.006
	Lpl	Lipoprotein lipase	-2.7	0.002
	Scd1	Stearoyl-Coenzyme A desaturase 1	5.8	0.003
**Cholesterol metabolism/pathway**				
	Abca1	ATP-binding cassette, sub-family A (ABC1), member 1	-1.5	0.010
	Cyp2e1	Cytochrome P450, family 2, subfamily e, polypeptide 1	-2.3	0.001
	Nr1h2	Nuclear receptor subfamily 1, group H, member 2	-1.6	0.024
	Nr1h4	Nuclear receptor subfamily 1, group H, member 4	-1.4	0.045
	Srebf2	Sterol regulatory element binding factor 2	-1.4	0.021
**Oxidative phosphorylation**				
	Ppa1	Pyrophosphatase (inorganic)	-1.6	0.008
**Adipokine Signaling**				
	Adipor1	Adiponectin receptor 1	-1.6	0.010
	Rbp4	Retinol binding protein 4	-1.5	0.005
**Insulin Signaling**				
	Pklr	Pyruvate kinase (liver)	2.1	0.003
	Igf1	Insulin-like growth factor 1	-1.7	0.011

Table S2. Cytokine levels in LPS challenged rats (n=4 per group).

Treatment group	TNF (ng/ml) 1 hour post LPS challenge	IL-6 (ng/ml) 1 hour post LPS challenge
**Vehicle**	7.43 (0.65)	23.9 (2.1)
**Dexamethasone (1 mg/kg)**	3.27 (1.50)*	13.7 (6.4)
**Dexamethasone (0.02 mg/kg)**	4.18 (1.61)	17.5 (5.7)
**Anti-CD163-dexamethasone (0.02 mg/kg)**	0.64 (0.33)**	7.0 (4.8)**
**Control IgG-dexamethasone (0.02 mg/kg)**	5.18 (1.18)	17.4 (3.9)
**Anti-CD163**	7.45 (0.96)	30.0 (0.6)

Values are means ±SEM. Significant differences between the vehicle group and the treatment groups are indicated by *$p<0.05$, **$p<0.01$.

Table S3. Food intake in treatment groups during the NASH treatment study.

Treatment group	HrF intake (g/day/cage*)
**Vehicle**	35.76 (1.03)
**Dexamethasone (0.02 mg/kg)**	35.40 (0.94)
**Anti-CD163-dexamethasone (0.02 mg/kg)**	35.40 (0.79)
**Control IgG-dexamethasone (0.02 mg/kg)**	36.00 (0.77)

Values are means ±SEM. The food intake was monitored during the entire experiment, but no significant differences were observed between the treatment groups. * Two rats per cage.

Table S4. Liver RNA profiling of genes significantly up- or down-regulated above a 1.4 fold threshold level in a treatment group(s) compared to the vehicle group. Fructose-induced up (↑) or down (↓)-regulation of those genes are indicated in the right side column.

Treatment group			Dexa	Anti-CD163-dexa	IgG-dexa	HFr-induced change
**Pathway/ response**	Gene symbol	Encoded protein	Fold Regulation	Fold Regulation	Fold Regulation	Up-or down Regulation
**Carbohydrate metabolism**						
	Gk	Glycerol kinase	1.5*	1.7*	1.9**	↓**
**Lipid and fatty acid metabolism**						
	Acly[A]	ATP citrate lyase	1.2	-1.4*	-1.2	↑**
	Acsm3	Acyl-CoA synthetase medium-chain family member 3	-1.8*	-1.3	-1.0	↓**
	Slc27a5	Solute carrier family 27, member 5	-1.1	1.3*	1.5**	↓*
**Cholesterol metabolism/transport**						
	Apoa1	Apolipoprotein A-I	1.4	1.3	1.5*	↓*
	Ldlr	Low density lipoprotein receptor	1.2	1.2	1.4*	NS
	Nr1h3	Nuclear receptor subfamily 1, group H, member 3	-1.2	-1.4**	-1.3	NS
	Ppara[A]	Peroxisome proliferator activated receptor-α	1.0	1.3	1.6**	↓**
	Ppard[A]	Peroxisome proliferator activated receptor-δ	1.2	-1.3	-1.9**	NS
	Pparg[A]	Peroxisome proliferator activated receptor-γ	-1.6*	-1.4	-13.5**	↓**
**Mitochrondial β-oxidation**						
	Acadl	Acetyl-Coenzyme A dehydrogenase, long-chain	1.3	1.6***	1.3	↓*
	Cpt1a	Carnitine palmitoyltransferase 1a, liver	1.5	2.4***	2.8**	↓*
**Adipokine signaling**						
	Lepr[A]	Leptin receptor	1.3	1.5**	1.6**	NS
**Insulin Signaling**						
	Insr	Insulin receptor	1.2	1.3	1.4*	↓**
	Ptpn1	Protein tyrosine phosphatase, non-receptor type 1	-1.1	-1.3	-1.7*	NS
**Inflammation**						
	Cebpb[A]	CCAAT/enhancer binding protein (C/EBP), β	-1.1	-2.1**	-2.1**	NS
	CD163	Hemoglobin scavenger receptor, CD163	-1.026	-1.236	-1.6*	↓*
**Apoptosis/Fibrogenesis**						
	Casp3	*Caspase 3, apoptosis-related cysteine protease*	1.4	1.5***	1.5**	NS
	Mapk8[A]	*Mitogen-activated protein kinase 8*	1.8	1.8*	1.8	NS
	Ifng[A]	*Interferon-γ*	-38.1*	21.6	-14.3	NS
	Serpine[A]	*Serine peptidase inhibitor, clade E, member 1*	-4.8	-5.9**	-3.3*	NS

Values are means ±SEM (n=8 per group). Significant differences are indicated by *p<0.05, **p<0.01-0.001. [A] Genes involved in several pathways/responses. NS indicates non-significant regulation.

Supplementary Material and Methods

The rat LPS model.
The experimental procedures were performed as described (27).

Fluorescence microscopy.
The experiment was performed as described in MATERIAL and METHODS (fluorescence microscopy).

RNA Profiling.

Total RNA was purified from liver using the RNeasy Mini Kit (Qiagen, København Ø, Denmark). A RT² Profiler™ PCR Array with 92 NASH biomarkers was designed for Fluidigm® BioMark™ analysis (see Supplementary Excel file for target and primer details). Reverse-transcription of RNA and preamplification of genes of interest was done using the Fluidigm Reverse Transcription Master Mix and the Fluidigm PreAmp Master Mix (Fluidigm Corporation) in accordance with the manufactures instructions. In short, for initial cDNA synthesis 25 ng of RNA was mixed with the RT mastermix (5x) in a 1:1 ratio and diluted 5 times in RNAse-free H_2O followed by incubation at the following conditions: 25°C for 5 min, 42°C for 30 min and finally 85°C for 5 min. To increase sensitivity, genes were pre-amplified by pooling 96 deltagene assays (Fluidigm, see Supplementary Excel for full list of target and primer details) allowing a final concentration of 500 nM per assay. In the final pre-amplification reaction mixture cDNA was diluted 1:4 with Fluidigm PreAmp Master Mix (5X), pooled deltagene assays (10X) and H_2O. Reaction mixture was incubated at the following PCR conditions: 95°C for 2 min followed by 14 cycles of 95°C for 15 sec and 60°C for 4 min. To remove unincorporated primers pre-amplified cDNA was incubated with 1,25U/µl Exonuclease I at 37°C for 30 min followed by 80°C for 15 min. After Exonuclease I treatment pre-amplified cDNA was diluted 1:5 with TE buffer (10 mM Tris-HCl, 1 mM disodium EDTA, pH 8.0) and stored at -20°C. Gene expression analysis was carried out using the 96.96 dynamic arrays and Biomark HD system from Fluidigm (Fluidigm Europe B.V.). Specifically, a 6 µl sample mixture was prepared for each sample containing 1x SsoFast EvaGreen Supermix with low ROX (BioRad), 1x DNA Binding Dye Sample Loading Reagent and each of diluted pre-amplified samples. Six µl of Assay mix was prepared with 1x Assay Loading Reagent, 100 µM of each of the different Deltagene Assays and TE buffer. The 96.96 dynamic arrays were primed and loaded in an IFC controller with in between additions of samples and assay mixes in the appropriate inlets. After loading, the chip was placed in the BioMark HD Instrument for an initial thermal mix that included incubation at 70°C for 40 min followed by 60°C for 0.5 min. Subsequent qPCR was performed by 1 cycle of hot start at 95°C for 1 min, followed by 30 cycles at 96° C for 5 sec and 60°C for 20 sec with fluorescent recording after each cycle. For analysis of PCR products the qPCR run was followed by a melting curve analysis where the fluorescent signal from dsDNA was measured at 60°C-95°C with 1°C increment. The data was analyzed with Real-Time PCR Analysis Software (Fluidigm Europe B.V.). The RT2 Profiler PCR Array Data Analysis Webportal (www.sabioscience.com) was used to analyze CT values and to calculate changes in gene expression. The relative expression levels of each target gene was normalized to ribosomal protein L13A and beta-actin and calculated by the 2-delta delta Ct method.
